# Genetic and physical interactions reveal overlapping and distinct contributions to meiotic double-strand break formation in *C. elegans*

**DOI:** 10.1101/2024.02.23.581796

**Published:** 2024-02-28

**Authors:** Marilina Raices, Fabiola Balmir, Nicola Silva, Wei Li, McKenzie K. Grundy, Dane K. Hoffman, Elisabeth Altendorfer, Carlos Jaime Camacho, Kara A. Bernstein, Monica P. Colaiácovo, Judith Yanowitz

**Affiliations:** 1 Magee-Womens Research Institute, Pittsburgh, PA 15213 USA; 2 Tsinghua U. Medical School, China; 3 Department of Biology, Masaryk University, Czech Republic; 4 Department of Microbiology and Molecular Genetics, University of Pittsburgh School of Medicine, UPMC Hillman Cancer Center, Pittsburgh, Pennsylvania; 5 Department of Genetics, Blavatnik Institute, Harvard Medical School, 77 Avenue Louis Pasteur, Room 334, Boston, MA 02115, USA; 6 Department of Computational and Systems Biology, University of Pittsburgh School of Medicine, Pittsburgh, PA, 15213 USA; 7 Department of Biochemistry and Biophysics, University of Pennsylvania, Penn Center for Genome Integrity, Philadelphia, Pennsylvania; 8 Department of Obstetrics, Gynecology and Reproductive Sciences, University of Pittsburgh School of Medicine, Pittsburgh, PA, 15213 USA.

**Keywords:** meiosis, double-strand break, *C. elegans*, SPO11, Crossover

## Abstract

Double-strand breaks (DSBs) are the most deleterious lesions experienced by our genome. Yet, DSBs are intentionally induced during gamete formation to promote the exchange of genetic material between homologous chromosomes. While the conserved topoisomerase-like enzyme Spo11 catalyzes DSBs, additional regulatory proteins—referred to as “Spo11 accessory factors”— regulate the number, timing, and placement of DSBs during early meiotic prophase ensuring that SPO11 does not wreak havoc on the genome. Despite the importance of the accessory factors, they are poorly conserved at the sequence level suggesting that these factors may adopt unique functions in different species. In this work, we present a detailed analysis of the genetic and physical interactions between the DSB factors in the nematode *Caenorhabditis elegans* providing new insights into conserved and novel functions of these proteins. This work shows that HIM-5 is the determinant of X-chromosome-specific crossovers and that its retention in the nucleus is dependent on DSB-1, the sole accessory factor that interacts with SPO-11. We further provide evidence that HIM-5 coordinates the actions of the different accessory factors sub-groups, providing insights into how components on the DNA loops may interact with the chromosome axis.

## INTRODUCTION

One of the seminal events during meiosis is the formation of crossovers (CO) during the first meiotic division. While COs create new combinations of alleles through the exchange of genetic material between homologs, they also establish physical connections that ensure their proper alignment on the meiotic spindle and their subsequent segregation to opposite ends of the spindle. In the absence of COs, homologs segregate randomly, leading to gametes with altered chromosome numbers and subsequent fetal aneuploidies. In humans, aneuploidy is observed in 1 of 160 live births (Dungan, 2002) and in >50% of miscarriages (Hassold et al., 2007) underscoring the importance of proper CO formation.

A necessary early step in CO formation is the creation of DNA double-strand breaks (DSBs) catalyzed by the widely conserved Spo11 enzyme (Bergerat et al., 1997; Keeney et al., 1997). Although Spo11 is essential for DSB formation, it does not function alone: additional proteins regulate the timing, placement, and number of DSBs (Cole et al., 2010; Hinman et al., 2021; Keeney, 2008). In *Saccharomyces cerevisiae*, where DSB formation has been best characterized, at least nine other proteins interact with Spo11 to regulate its recruitment and activation (Lam & Keeney, 2015; Martini et al., 2006). *In vivo* and *in vitro* physical interaction studies have revealed that these proteins form several subcomplexes. Briefly, the RMM complex (Rec114, Mer2, and Mei4) is loaded on chromosomes early in prophase and is required for Spo11 binding to sites of DNA cleavage (J. Li et al., 2006; Sasanuma et al., 2007). Activation of Mer2 requires phosphorylation by cyclin-dependent kinase (CDK) (Henderson et al., 2006), suggesting that Mer2 links DSB formation to the progression of the meiotic program (Arora et al., 2004; Henderson et al., 2006; J. Li et al., 2006; Maleki et al., 2007). Rec102 and Rec104 form another subcomplex that facilitates Spo11 dimerization and its association with DSB sites (Arora et al., 2004; Kee & Keeney, 2002; Prieler et al., 2005; Sasanuma et al., 2007). Ski8 binds Spo11 and is important for Spo11 nuclear localization (Arora et al., 2004). Finally, the Mre11–Rad50–Xrs2 (MRX) complex is also required for DSB formation but requires all the other DSB proteins to associate with DSB sites. Despite their importance, most of these proteins are poorly conserved at the amino acid level although homologs have been identified for several of these in organisms ranging from plants to worms to mice (Cole et al., 2010; Kumar et al., 2010). While a recent study of plants has presented a similar model for DSB induction as in yeast (Vrielynck et al., 2021), our understanding of the DSB machinery in metazoans is lacking.

*In vitro* biochemical studies have shown that *C. elegans* SPO-11 is monomeric and, even when it is able to bind dsDNA, it does not exhibit DNA cleavage activity (Yeh et al., 2017). This observation strongly supports the hypothesis that cofactors are needed for DSB induction. Indeed, *dsb-1*, *dsb-3*, *him-17*, *mre-11*, and *rad-50* are all essential for the introduction of breaks in this organism (Chin & Villeneuve, 2001; Hayashi et al., 2007; Hinman et al., 2021; Reddy & Villeneuve, 2004; Stamper et al., 2013). Other genes, including *xnd-1*, *him-5, rec-1, dsb-2, cep-1,* and *parg-1* have more limited roles in break induction, with the former two genes required to ensure the full complement of DSBs on the X chromosome (Janisiw et al., 2020; Meneely et al., 2012; Rosu et al., 2013). *xnd-1* is also unique among the accessory factors in influencing the timing of DSBs; in the absence of *xnd-1*, there is precocious and rapid accumulation of DSBs as monitored by the accumulation of the HR strand-exchange protein RAD-51(Gao et al., 2015; McClendon et al., 2016; Wagner et al., 2010). CO positioning is altered in all situations where DSB levels are affected, but the *rec-1* mutation has a profound effect on the distribution of meiotic COs (Rose & Baillie, 1979) with only a minor reduction in total DSB numbers (Chung et al., 2015).

Prior analysis of *rec-1* and *him-5* found these genes to be paralogs that function cooperatively to ensure wild-type DSB levels (Chung et al., 2015). *him-5* has also been shown to have synthetic interactions with *cep-1,* and epistatic relationships with *xnd-1* and *him-17*, in part because *xnd-1* regulates *him-5* transcriptionally (McClendon et al., 2016). These studies also revealed roles for *him-5* in coordinating DSB induction with downstream repair.

In this study, we expand the analysis of the genetic interactions between the SPO-11 accessory factors in *C. elegans* and provide the first biochemical studies of protein-protein interactions between these factors by co-immunoprecipitation and support of yeast-two hybrid (Y2H). We propose for the first time a possible complex that regulates DSB formation in *C. elegans* and compare and contrast this to models recently proposed in yeast and plants (Bouuaert et al., 2021, Vrielynck et al., 2021). Our results allow us to begin to elucidate the regulatory events that control the localization, timing, and placement of meiotic DSBs in *C. elegans*.

## RESULTS

### HIM-5 is the essential factor for meiotic breaks in the X-chromosome

**Figure 1:**
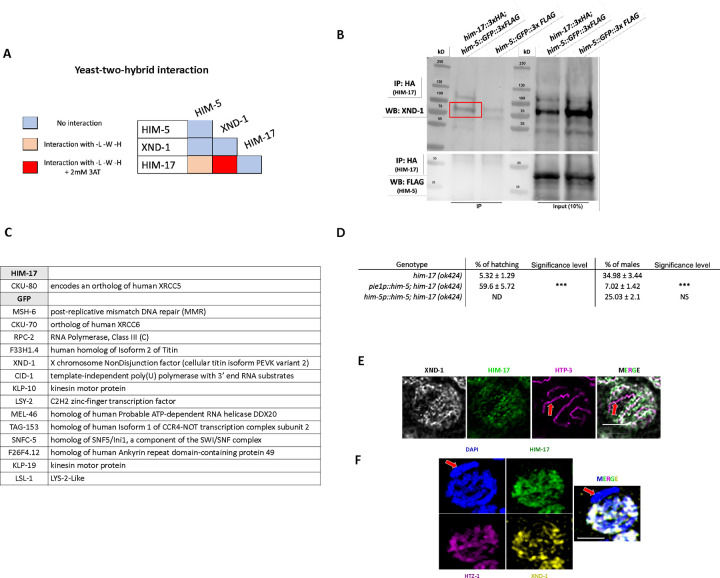
XND-1 and HIM-17 interact and are predominantly localized away from the DNA axis. **A)** Summary of the yeast two-hybrid assay results. The complete set of results is given in Figure S1A. **B)** Western blot analysis of endogenous XND-1 (top) and HIM-5::GFP::FLAG (bottom) on HA pull-downs performed in *him-17::3xHA;him-5::GFP::3xFLAG* strain*.* Analysis was performed in biological duplicates. **C)** Mass spectrometry results of GFP pulldowns of HIM-17::GFP. IP/MS analysis was performed in duplicate and corrected for nonspecific binding of proteins bound to GFP trap beads. **D)** Quantification of embryonic viability (hatching rates) and frequencies of male offspring among the progeny of the indicated genotypes. Data are shown as mean +/− SD; NS stands for not significant; **** p < 0.0001, n= number of P_0_ parents whose brood was examined. **E)** Representative STED images of mid-pachytene stage nuclei showing substantial overlap of XND-1 and HIM-17 on DNA not at the chromosome axis (stained with HTP-3). The X chromosome is not stained by either XND-1 and HIM-17 (red arrow). **F)** Representative confocal images showing co-localization of XND-1 and HIM-17 with an autosome-enriched variant histone, HTZ-1. Red arrow indicates the X chromosome. Scale bars = 2μm.

Three of the DSB proteins appear to have a biased effect on X chromosome CO formation: null alleles of *xnd-1* and *him-5* predominantly show a loss of CO intermediates on the X, increases in univalent X chromosomes at diakinesis in half or almost all nuclei, respectively, and an increase in XO male offspring resulting from nondisjunction of the X chromosome during the meiotic division; the hypomorphic *him-17(e2806)* mutations shows increased incidence of one unattached homolog pair in diakinesis and an increase in male production consistent with an X-specific defect (Reddy & Villeneuve, 2004). To determine if these genes function through a common underlying mechanism, we first performed yeast two-hybrid (Y2H) assessing interactions with both high and low stringency. We observed a strong Y2H interaction between XND-1 and HIM-17, and a weak one between HIM-17 and HIM-5 (Figure 1A). To validate these interactions, we also performed immunoprecipitations (IPs) of HIM-17 followed by mass spectrometry (MS). This identified XND-1, as well as 16 additional strong interactors of HIM-17 (Figure 1B). Moreover, endogenous XND-1 was identified in western blots of IPs performed independently using 3xHA-tagged HIM-17 epitopes, indicating a robust interaction between these proteins (Figure 1C). In contrast, HIM-5 was not detected in either the HIM-17 co-IPs or IP/MS, suggesting that the interaction observed by Y2H either exists very transiently or was bridged by a protein expressed in yeast.

Prior studies from our group indicate that *xnd-1* regulates HIM-5 transcriptionally since ectopic expression of *him-5::GFP* using the *pie-1* germline promoter, but not expression using the endogenous *him-5* promoter, rescues the X chromosome nondisjunction defects conferred by the *xnd-1* mutation (Wagner et al., 2010, Meneely et al., 2012; McClendon et al., 2016). Since *xnd-1* and *him-17* both function to regulate X chromosome DSBs and their encoded proteins physically associate (Figure 1A–C), we reasoned that *him-17* might also regulate *him-5* expression. As seen in *xnd-1* mutants, ectopic expression of *Ppie-1::him-5* in *him-17* null mutants rescued the X chromosome defects seen by the increase of XO male offspring from XX hermaphrodites (known as the high incidence of males or “Him” phenotype) (34.98% of males in *him-17(ok424)* vs. 7.02% in *pie1p::him-5;him-17(ok424)*) (Figure 1D). When *him-5* was expressed from its own promoter (*Phim-5::him-5*), there was only a minor suppression of both the Him phenotype and hatching rates (Figure 1D) and almost no change in univalent formation (Figure S2) despite higher HIM-5 expression in the *Phim-5::him-5* strain than in the *Ppie-1::him-5* strain (McClendon et al., 2016). Thus, we infer that XND-1 and HIM-17 both regulate the expression of HIM-5 to promote X chromosome DSB/CO formation. In addition, *xnd-1* and *him-17* have independent roles in DSB/CO formation since only null alleles of *him-17*, but not *xnd-1* or *him-5*, confer a severe loss of DSBs (Meneely et al., 2012; Reddy & Villeneuve, 2004; Wagner et al., 2010); and only *xnd-1* mutations affect the timing of DSBs/repair (Wagner et al., 2010; McClendon et al., 2016).

We note that ectopic HIM-5 expression strongly rescued the embryonic lethality conferred by the *him-17* null allele. While a small fraction of the lethality is attributed to X chromosome nondisjunction, most results from missegregation of the autosomes. Thus, even though *him-5* null mutant animals exhibit a profound loss of DSBs/COs on the X chromosomes with rare CO defects on autosomes (Meneely et al., 2012), *him-5* expression can restore CO formation to both autosomes and X chromosomes in the *him-17* mutant background. This strongly supports a general role for HIM-5 in CO formation and further indicates that HIM-5 is sufficient to restore COs on the X chromosome.

To further characterize the interaction between XND-1 and HIM-17, we also sought to determine whether they colocalize on meiotic chromosomes using super-resolution microscopy. Prior studies have shown that HIM-17 associates with meiotic chromatin (Reddy & Villeneuve, 2004). Here we provide evidence that HIM-17 is preferentially enriched on the autosomes, colocalizing with markers of active chromatin transcription. Co-staining the HIM-17::HA transgenic line with anti-HA and anti-XND-1 antibodies showed that both proteins predominantly localize off of the chromosome axes (marked with HTP-3), although some of each protein can be seen close to the axes (Figure 1E and F).

### DSB-1 mediates interactions with SPO-11

**Figure 2:**
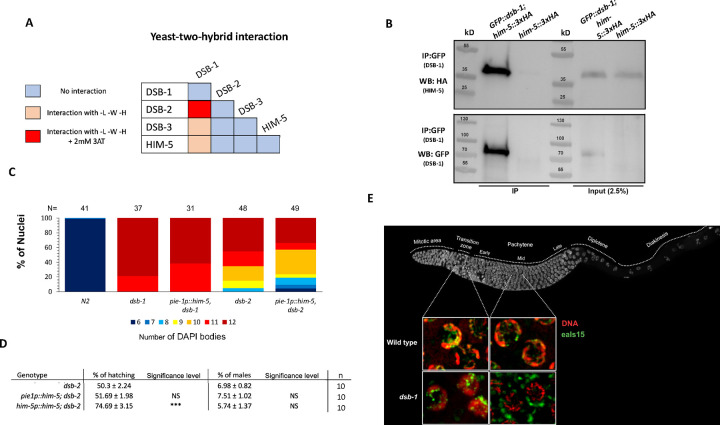
DSB-1 associates with HIM-5 and regulates its localization **A)** Summary of the yeast two-hybrid assay results. The complete set of results is given in Figure S1A. **B)** Western blot analysis of GFP pull-downs performed in *GFP::dsb-1;him-5::3XHA* strain showing co-IP of HA-tagged HIM-5 proteins*.* Analysis was performed in biological duplicates. **C)** Quantification of DAPI-stained bodies at diakinesis for the indicated genotypes. Colors correspond to the number of DAPI-stained bodies shown in the key below. **D)** Quantification of embryonic viability (hatching rates) and frequencies of male offspring among the progeny of the indicated genotypes. Data are shown as mean +/− SD; NS stands for not significant; **** p < 0.0001, n= number of P_0_ parents whose brood was examined. **E) Top:** *C. elegans* gonad fixed and stained with DAPI to show the organization and distribution of the nuclei along the Prophase I. **Bottom:** live images of nuclei in the transition zone (leptotene-zygotene) and middle-pachytene. *eaIs15 (Ppie-1::him-5)* is visualized in freshly dissected gonads by GFP fluorescence (green), and DNA by DRAQ5 (red). In *dsb-1* mutants, HIM-5 is located is nuclear in the transition zone and becomes located in cytoplasmic puncta by middle pachytene.

Since HIM-5 appears to be central to DSB/CO formation on the X chromosome and mutations in *dsb-1* and *dsb-3* are required for all meiotic breaks, we next sought to determine whether HIM-5 interacts with DSB-1, its paralog DSB-2, or DSB-3. We found that DSB-1 and DSB-2 show robust Y2H interactions and that DSB-1 and DSB-3 exhibit weak Y2H interactions (Figure 2A), confirming results from a recent study (Hinman et al., 2021). We also found that HIM-5 shows weak Y2H interactions with DSB-1 (Figure 2A). We confirmed the interaction between DSB-1 and HIM-5 with co-IP, pulling down GFP::DSB-1 and probing for HIM-5::3xHA by Western blotting (Figure 2B).

To begin to address the functional relevance of the interactions between DSB-1 and HIM-5, we asked whether ectopic expression of HIM-5 could suppress the defects in bivalent formation due to the loss of *dsb-1* or *dsb-2* functions. As shown in Figure 2C, ectopic HIM-5 very slightly reduced the number of univalents in either *dsb-1* or *dsb-2*. In *dsb-1* mutants. The increase in *dsb-1* is seen by the shift from 12 to 11 DAPI staining bodies (corresponding to 12 univalents and 1 bivalent and 10 univalents, respectively) in ~20% of diakinesis nuclei. A similar shift is seen in *dsb-2*, suggesting that HIM-5 provided one additional DSB/CO in these mutant backgrounds. Consistent with little to no suppression, *pie-1::him-5* did not suppress the hatching defects or male production in *dsb-2*. However, a slight suppression of the embryonic lethality is seen with the *him-5p::him-5* transgene which is more highly expressed (Figure 2D). The ability to suppress hatching but not the male production further supports the role of HIM-5 in regulating total DSB numbers.

The inability of ectopic HIM-5 to strongly suppress *dsb-1* suggests that, unlike XND-1 and HIM-17, DSB-1 does not regulate the expression of the *him-5* locus. However, using both live imaging of HIM-5::GFP transgenes or anti-HA staining for endogenously-tagged HIM-5::3xHA, we observed mislocalization of HIM-5 proteins in the *dsb-1* mutant background (Figure 2E). Interestingly, the initial localization of HIM-5 is normal with nuclear localization in the transition zone (leptotene/zygotene stage) nuclei. However, whereas in the wild type, HIM-5 protein remains nuclear throughout early-mid pachytene; in *dsb-1* mutants, HIM-5 localizes to cytoplasmic puncta at mid-pachytene. suggesting that DSB-1 (and/or its DSB-promoting function) is required to retain HIM-5 in meiotic nuclei.

### Genetic interactions define four epistasis groups that contribute to DSB/CO formation.

Having established interactions between subsets of the SPO-11 accessory factors in *C. elegans*, we next wanted to perform more extensive genetic analyses between the known DSB factors. To this end, we created double mutant combinations of alleles with partial DSB defects. These included either weak loss-of-function alleles of essential DSB genes (*him-17, mre-11*) or null alleles that reduce DSBs (*cep-1*, *dsb-2*, *rec-1*, *him-5*, and *parg-1*). Diakinesis oocytes were analyzed in the single and double mutants by whole-mount fixation and DAPI staining followed by confocal microscopy and 3D visualization. The chromosomes are highly condensed at diakinesis, and six bivalents can be detected in wild-type worms. Univalents, fusions, and DNA fragments can be seen in mutants with defects in DSB formation or their repair into a CO, respectively (Chin & Villeneuve, 2001; Guo et al., 2022; Rosu et al., 2013). We hypothesized that double mutant strains carrying mutations in genes that function in different aspects of DSB induction would show more severe defects and have an increase in univalent formation compared to single mutants. On the other hand, genes that function together or at the same step of break induction should exhibit no or only mild enhancement of the DSB defects.

**Figure 3:**
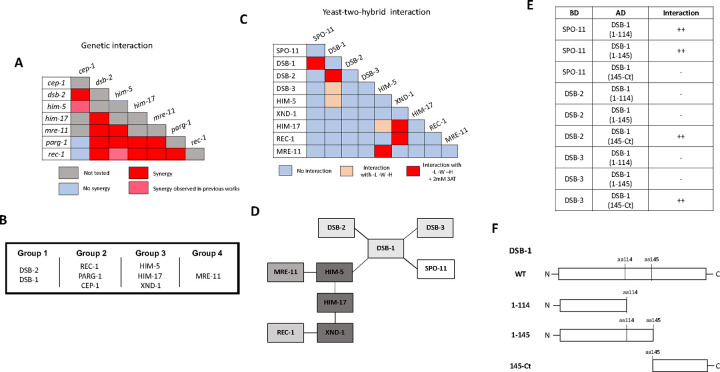
Genetic and protein-protein interaction studies show evidence of DSB protein sub-complexes in *C. elegans.* **A)** Summary of the genetic interaction results. The complete set of results is given in Figure S3. **B)** Genetic groups are based on data presented here and previously published (Chung et al., 2015; Mateo et al., 2016; McClendon et al., 2016; Janisiw et al., 2020). **C)** Summary of the yeast two-hybrid assay results. The complete set of results is given in Figure S1A. **D)** Schematic representation of the interaction network based on Y2H interactions. Thick line: Strong interaction between two proteins. Thin line: weak interaction between two proteins. **E)** Different DSB-1 constructs (detailed in F) were used to analyze yeast-two-hybrid interactions with SPO-11, DSB-2, and DSB-3. **F)** Scheme representation of the wild-type DSB-1 structure, and the different truncations used in **E)** For more details about the DSB-1 sequence, protein structure, and deletions used in these experiments, see Figure S5.

Genetic epistasis was precluded in *dsb-1* and *dsb-3* since the single mutants already show a complete absence of breaks (Hinman et al., 2021; Stamper et al., 2013). By contrast, *dsb-2* mutant nuclei manifest an age-dependent loss in DSB capacity (Rosu et al., 2013; Stamper et al., 2013) allowing us to analyze interactions between *dsb-2* and *cep-1, parg-1, him-17,* and *rec-1* (Figure 3A and Figure S3A–D). As 1-day-old adults, *dsb-2* single mutants had ~50% of diakinesis nuclei that contained 6 bivalents, ~30% with 7 DAPI-stained bodies (5 bivalents and 2 univalents), and ~20% with 8 or more DAPI-stained bodies. Single mutants of *cep-1*, *parg-1,* and *rec-1* each had >90% of diakinesis nuclei with 6 bivalents, consistent with prior studies (Chung et al., 2015; Janisiw et al., 2020; Mateo et al., 2016). In animals carrying *him-17(e2806)* mutation, a weak allele of the otherwise essential locus for DSB formation, only ~50% of mutant nuclei had 6 bivalents; the other 50% had 7 DAPI-stained bodies, reflecting the requirement for this gene in CO formation on the X chromosome (Reddy & Villeneuve, 2004). When *dsb-2* was combined with each of these mutations, an increased number of DAPI-stained bodies and a concomitant decrease in the number of chiasmata were observed. These results strongly suggest that *dsb-2* functions cooperatively with all four loci for the regulation of meiotic CO formation.

Given that *rec-1, parg-1,* and *cep-1* fall in a separate epistasis group from *dsb-2*, we next addressed whether these three genes function together or independently. All double mutant permutations of *rec-1, parg-1,* and *cep-1* were analyzed, and in each case, the single and double mutants were nearly indistinguishable from the single mutants, i.e., 6 bivalents (Figure S3L–M). Therefore, we infer that these genes fall into the same epistasis group and therefore likely function to control the same step in DSB/CO induction. Consistent with this interpretation, when *rec-1, parg-1,* or *cep-1* were combined with other DSB gene mutations, including *him-17*, they had similar effects, and the numbers of univalents were increased (Figures S3A, C, D for Group 1; Figure S3E–G for Group 3; and Figure S3J, K for Group 4). Similar results have previously been reported from our laboratories for double mutants with *him-5* (Chung et al., 2015; Janisiw et al., 2020; Mateo et al., 2016).

*mre-11* functions in both DSB formation and subsequent DNA end resection (Chin & Villeneuve, 2001), the latter as part of the MRE11-RAD50-NBS1/Xrs2 (MRN/X) resection complex, the other proteins of which are encoded by the worm *rad-50* and *nbs-1* genes (Girard et al., 2018; Hayashi et al., 2007). Null mutations in *mre-11* and *rad-50* completely abrogate DSB induction. However, we were able to take advantage of a separation-of-function allele, *mre-11(iow1),* that cannot perform its end-resection functions but can induce DSBs (Yin & Smolikove, 2013). The *iow1* mutation causes chromosome fusions to appear in diakinesis oocytes resulting from the aggregation of unprocessed DNA ends. While this allele makes enough DSBs to cause aggregation of all chromosomes, we hypothesized that it might have a minor impairment in DSB induction that would be revealed when combined with other DSB mutations. If this were the case, the double mutants would make fewer breaks, leading to fewer chromosome fusions and an increase in the number of DAPI-stained bodies/univalents. Consistent with this hypothesis, *mre-11(iow1)* double mutants with each of the other DSB mutations led to fewer fusions and more DAPI-stained bodies (Figure S3H–K; Figure 3A). Thus, we propose that *mre-11,* and by extension *rad-50*, function in their own branch of the DSB/CO pathway.

Surprisingly, a unique phenotype was observed in a subset of the *cep-1; mre-11* double mutants*.* Our prior studies showed that *cep-1* functions redundantly with *him-5* to both ensure DSB formation and promote downstream homologous recombination (HR) repair by preventing non-homologous end joining (NHEJ) (Mateo et al., 2016). In that study, we reported that *cep-1(lg12501)* is a separation-of-function allele that is defective in DSB formation. In the *cep-1(lg12501);mre-11(iow-1)* double mutants, we observed mixed phenotypes: some nuclei had 12 DAPI-stained bodies, while others exhibited an exacerbation of the fusion defect (Figure S4). We infer that the univalents indicate a synergistic effect between *mre-11* and *cep-1* for DSB formation; the increase in fusions, on the other hand, may suggest that *cep-1(lg12501)* is not fully wild-type for the DNA repair functions of CEP-1.

While each of the genes that we analyzed has reported roles in DSB formation, we wanted to confirm that the observed increases in univalents in the double mutants were in fact due to impairment in DSB induction and not to deficits in later steps of CO formation. Therefore, we relied on an established assay to distinguish between these possibilities: we asked whether the addition of exogenous breaks from gamma irradiation (IR) could suppress the univalent phenotype by serving as a surrogate for SPO-11-induced DSBs (Dernburg et al., 1998). Recent work has shown that 10Gy IR is able to produce up to ~20 DSBs per meiotic nucleus (Lascarez-Lagunas et al., 2023), which is sufficient to ensure a CO on each chromosome (Mets & Meyer, 2009). We analyzed the number of DAPI bodies with and without IR in a subset of the double mutants where we observed synergistic effects (*dsb-2;parg-1*, *dsb-2;rec-1*, and *rec-1;him-17*). When each of these double mutant strains was exposed to IR, the wild-type phenotype of 6 bivalents was largely restored (Figure S5), supporting the conclusion that the CO deficiency observed is triggered by defects in DSB formation.

### Protein-protein interactions between the DSB regulatory factors

The genetic analyses described above defined four epistasis groups (Figure 3B). Using Y2H, we next set out to determine whether interactions between groups could explain how the different complexes come together to regulate DSB induction (Figure 3C–D). CEP-1 showed auto-activation in both vectors and therefore was removed from further analysis. DSB-1, HIM-17, and REC-1 also showed self-activation in the binding domain vector, therefore, only the activation domain fusions were analyzed. Pairwise interactions between all remaining combinations were then analyzed. HIM-5 displayed the most promiscuous Y2H interactions, with strong interactions seen with MRE-11 and weak interactions as already discussed with HIM-17 and DSB-1. XND-1 apart from interacting with HIM-17, also showed strong Y2H interactions with REC-1. A recent study provided Y2H evidence supporting the idea that SPO-11 interacts with DSB-1 (Hinman et al., 2021). Critically, our work supports and extends this observation to show that DSB-1 is the only known accessory factor that interacts with SPO-11 in Y2H assays, cementing its role as a key determinant for DSB induction and explaining its role in feedback controls (Stamper et al., 2013; Hinman et al., 2021; Guo et al., 2022).

To analyze more in-depth the Y2H interactions between DSB-1 and DSB-2, DSB-3, and SPO-11, we looked for protein domains in DSB-1 that could be involved in these interactions. Interestingly, DSB-1 has 5 “sticky” helices (helices 2–6 in Figure S6) that have the potential to be implicated in protein-protein interactions. We first deleted all of these helices together by introducing a deletion after L114 (DSB-1(1–114) in Figures 3E, F). We found that this truncated version of DSB-1 loses the ability to interact with DSB-2 and DSB-3 but is still able to associate with SPO-11. Moreover, when we only deleted the helices 3–6 but kept the helix 2 (DSB-1(1–145)), we obtained the same results, suggesting that the Y2H interactions between DSB-1 and DSB-2/DSB-3 is mediated by the C-terminal helices 3–6 and not helix 2. To prove this, we created a truncated DSB-1 protein that only contains amino acids from P145 to the stop codon (DSB-1(145-Ct)). Consistent with the deletion analysis, we observed that the C-terminal domain is sufficient to promote associations with DSB-2 and DSB-3. This domain was also not sufficient to promote the DSB-1/SPO-11 interaction (Figure 3E). These experiments complement the results published by Villeneuve lab where they observed that the DSB-1 N-terminus is the domain necessary for the DSB-1/SPO-11 (but not for the DSB-1/2/3) interaction (Hinman et al., 2021). Together, these studies support the idea that the DSB-1/SPO-11 and the DSB-1/2/3 interactions are mediated by distinct domains of DSB-1.

## DISCUSSION

**Figure 4:**
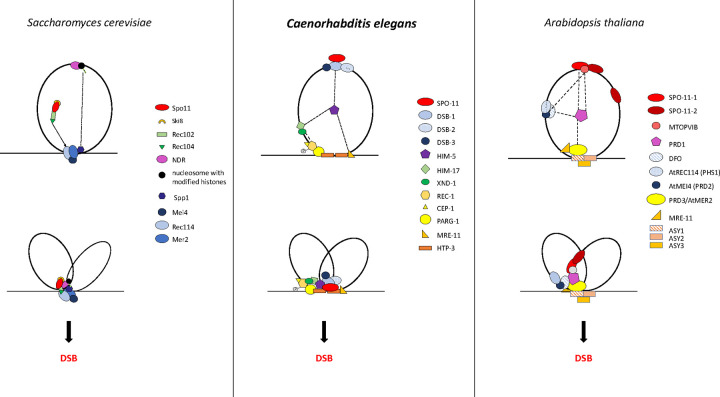
Model of the DSB formation complex compared in *S. cerevisiae*, *C. elegans,* and *A. thaliana.* The results of this paper and previous work discussed in this article allow us to propose a model to explain the known interactions and localization patterns of the DSB regulatory factors in *C. elegans* (center) where SPO-11 forms a subcomplex with DSB-1/−2/−3 that is located initially on the DNA loops and that we propose is brought to the chromosome axes by association with HIM-5 and its binding to MRE-11 and PARG-1 which both associate with HTP-3. SPO-11 could also be recruited to the DSB-1 after axis association. Other actors of the complex are also initially on the chromatin loops and also get recruited to the axes by HIM-5, allowing the formation of DSBs and coupling of DSBs to downstream repair by the MRN complex and others. This model shares some similarities to what was proposed for *S. cerevisiae* (Bouuaert et al., 2021b; Sommermeyer et al., 2013)and *A. thaliana* (Vrielynck et al., 2021). For more details see the Discussion.

Across species, accessory factors collaborate with SPO-11 to regulate various aspects of DSB formation, including the timing, placement, number, and persistence of DSB induction, as well as the chromatin architecture at and around the break site (Arora et al., 2004; Cole et al., 2010; Hinman et al., 2021; Kumar & de Massy, 2010; Panizza et al., 2011; Sasanuma et al., 2007; Sommermeyer et al., 2013; Vrielynck et al., 2021). Here were present genetic, biochemical, and cytological studies to develop the first comprehensive model for DSB regulation in a metazoan. The *C. elegans* DSB complex appears to be comprised of four sub-groups (Figure 3B and 4) interconnected by interactions with HIM-5. Our results suggest two important roles for HIM-5: first as the linchpin between the whole DSB complex, thereby ensuring the coordination of the timing, placement, and number of DSBs (described in more detail below); and second, as the determinant in X chromosome COs.

DSB-1 and DSB-2 are distant paralogs, and they are both orthologs of the budding yeast Rec114 protein (Rosu et al., 2013; Stamper et al., 2013). On the other hand, DSB-3 is an ortholog of yeast Mei4 (Hinman et al., 2021). In yeast and *Arabidopsis,* accessory proteins are associated indirectly with Spo11/SPO11 through interactions with the TopVI/MTOPVIB subunit (Arora et al., 2004; Kee & Keeney, 2002; Maleki et al., 2007; Robert et al., 2016; Vrielynck et al., 2021). These differences between species underscore how conserved DSB proteins have been co-opted to regulate different aspects of break induction. The lack of direct association between SPO-11 and the other worm accessory factors points to DSB-1 as a hub for the integration of various signals regulating break induction. For example, DSB-1 and DSB-2 persist longer on chromatin in strains that are defective in DSB induction (e.g. *mre-11; him-17*), DNA repair, and SC formation. Their persistence has been suggested to be required to maintain SPO-11 in a DSB-permissive state (Rosu et al., 2013; Stamper et al., 2013). This was supported by studies that both identified ATM/ATR binding sites on these proteins (W. Li & Yanowitz, 2019) and showed that phosphorylation of DSB-1 retains break competency (Guo et al., 2022). Together, these data suggest that DSB-1 is responsible for inducing a second wave of DSBs mediated by activation of ATL-1/ATM-1.

While DSB-1/-2/-3 are critical for CO formation, they predominantly localize on DNA loops with little to no overlap (Hinman et al., 2021). Instead, a small fraction of these three proteins appear to associate with the DNA axis, a localization that is consistent with the “tethered loop model” for break induction in yeast and plants (Kee et al., 2004; Maleki et al., 2007; Panizza et al., 2011; Sommermeyer et al., 2013; Tsai et al., 2020). Two accessory factors, MRE-11 and PARG-1 are known interactors of the axis protein HTP-3 (Janisiw et al., 2020). Both proteins interact either directly or indirectly with HIM-5, which physically associates with DSB-1, thereby providing a mechanism to bring the loop-associated proteins to the axis.

MRE-11 and PARG-1 also have roles in both break formation and DNA repair. For PARG-1, we show here that it functions together with REC-1 and CEP-1 based on the following data: synthetic CO defects were seen in *cep-1;him-5*, *rec-1;him-5*, and *parg-1;him-5* (Chung et al., 2015; Mateo et al., 2016 and this paper), but not in *rec-1;cep-1, rec-1;parg-1*, and *cep-1;parg-1* double mutants; *dsb-2* mutant phenotypes are exacerbated by the loss of *rec-1*, *cep-1* (Figure S3), and *parg-*1 (Janisiw et al., 2020). Interestingly, both CEP-1 and PARG-1 have additional roles in promoting homologous recombination-mediated repair and/or preventing alternative repair pathways (Janisiw, et al., 2020; Mateo et al., 2016). Therefore, we envision that CEP-1, REC-1, and PARG-1 function together to promote DSBs and their repair into COs at the chromosome axis. Since we also showed that the DSB defects in *mre-11* mutant animals were enhanced by loss of *parg-1* or *rec-1* (Figure S3), we favor a model in which MRE-11 and PARG-1 independently interact with the chromosome axis to ensure robust DSB formation and/or repair. It is possible that MRE-11, which is essential for COs, is the major pathway for tethering the loops to the axis, but further structural studies of these complexes are required.

The defects observed in *cep-1;him-5* (Mateo et al., 2016), and *rec-1;him-5* (Chung et al., 2015) are both exacerbated by maternal age, similar to *dsb-2*. We envision that inputs from both HIM-5 and DSB-2 converge on DSB-1 to ensure robust DSB formation across the lifespan. This would also explain the synthetic defects in DSB formation between *dsb-2* and each of the components in the *him-5* branch.

## TIMING of BREAKS

Studies from multiple systems have elucidated mechanisms to coordinate the timing of DSBs with the completion of meiosis, e.g. by CDK and Ddf4 phosphorylation in yeast (Murakami & Keeney, 2008). In worms, REC-1 was previously shown to be a target of CDK and therefore it was hypothesized that such a phosphorylation event would be critical to ensure that break induction occurs after the completion of S phase (Chung et al., 2015). REC-1 and HIM-5 evolved from a gene duplication event in the *C. elegans* lineage; whereas the ancestral gene in related nematodes has features of both proteins- although it shares greater homology with HIM-5 (Chung et al., 2015). Thus, we hypothesize that the single ancestral REC-1/HIM-5 protein would be subject to CDK phosphorylation, which would then promote the assembly of the complexes at the chromosome axis to promote break formation.

Timing also appears to be critical for CO outcomes. Studies in yeast show different outcomes for early and late breaks (Joshi et al., 2015) and recent studies in worms indicate that later breaks are needed for CO formation (Hicks et al., 2022). We previously showed that *xnd-1* mutants have altered DSB kinetics, showing elevated levels of DSB formation in leptotene/zygotene (McClendon et al., 2016; Wagner et al., 2010). This function of XND-1 is mediated, at least in part, by its effect on chromatin architecture. One possibility is that the physical interaction that we show here between XND-1 and REC-1 couples the completion of S phase to the activation of breaks within the correct chromatin environment, thus ensuring their timely formation. In the absence of XND-1, increases in global histone acetylation (Gao et al., 2015; Wagner et al., 2010) might allow SPO-11 easier access to the DNA. Another possibility is that upon phosphorylation of REC-1 by CDK-1, REC-1 through PARG-1 tethers XND-1 (the timer) to the axis, an event that restrains DSB formation.

### Chromatin and Meiotic Gene Transcription

We also provide evidence for physical interactions between XND-1 and HIM-17, both by Y2H and co-IP. HIM-17 appears to have a role in transcriptional regulation of DSB factors. Here we show that ectopic expression of HIM-5 from the *pie-1* promoter, but not overexpression from the endogenous *him-5* promoter, suppressed *him-17* phenotypes. This is similar to the rescue we saw of *xnd-1* (McClendon et al., 2016) and suggests that both proteins contribute to the expression of *him-5*. In addition, a recent study found that *him-17* regulates the expression of ~300 germline-enriched genes, including *him-5, rec-1*, and *dsb-2*, (Carelli et al., 2022) whose concomitant loss would be expected to abrogate break induction (Chung et al., 2015 and Figure 3A and Figure S3). While its only role may be in transcriptional regulation, HIM-17 also showed a weak physical interaction with HIM-5 in our Y2H assays. Although these interactions were not confirmed by IP, it remains possible that these proteins have transient associations that are unstable during IP or are not abundant. Together with observed changes in chromatin structure and both DSB and CO distribution in *him-17* mutants(Nadarajan et al., 2021; Reddy & Villeneuve, 2004), it is tempting to speculate that together with XND-1, HIM-17 helps to create an environment permissive for SPO-11 binding and/or break formation. XND-1 and HIM-17 may function together in this role or redundantly, perhaps through XND-1 interacting with REC-1 and HIM-17 interacting with HIM-5, or in related species through interactions with the REC-1/HIM-5 paralog.

### X/Autosome differences in DSB formation

One of the conundrums that our data presents is the function of the HIM-5 protein. Our Y2H and genetic data place this protein as a key mediator between the SPO-11-DSB complex and the other accessory factors. However, mutations in *him-*5 preferentially impact CO formation on the X chromosome (Broverman & Meneely, 1994; Hodgkin et al., 1979). Furthermore, *rec-1;him-5* double mutants give an age-dependent severe loss of DSBs (like *dsb-2* mutants) suggesting that the ancestral function of the protein may have a more profound effect on break formation. While the X-specific phenotypes conferred by *him-5* mutation could suggest that this chromosome is simply more sensitive to a reduction in the number of active SPO-11 complexes, the fact that mutations in *dsb-2* also reduce DSBs but without an X chromosome bias argues against this interpretation. HIM-5 must have additional roles beyond directly regulating the activation of SPO-11. We hypothesize that HIM-5 is the critical factor to ensure that breaks occur on the X chromosome. In support of this, we show here that ectopic expression of *him-5* can substantially rescue both the autosomal and X chromosomal defects of *him-17* null mutations despite expression levels lower than endogenous *him-5*(McClendon et al., 2016). The X chromosome-specific defects of *him-5* mutations might arise due to defects in recruitment of DSB-1–2-3/SPO-11 to specific sites (hotspots) on the X or to an impaired ability to coordinate the assembly of the DSB machinery on the axis of the X chromosome, although other models can be envisioned.

### Concluding Remarks

Our Y2H and IPs results show very strong protein-protein interaction between the different DSB factors, however, it is also a fact that their localization along the gonads does not always follow the same patterns. This can suggest that even when the proteins can interact, this interaction might be time-space regulated depending on the role that each of these proteins has during meiosis. Further studies are necessary to elucidate how these interactions arise and change *in vivo.*

## METHODS

### Culture and strains

Worms were cultured on MyoB plates (Burns et al., 2006)) seeded with OP50 at 20°C, unless otherwise noted (Brenner 1974). Mutant strains used in this study are listed in Supplemental Material, Table S1. All strains were derived from the wild-type Bristol strain N2.

### Chromosome morphology analysis of diakinesis oocytes

The numbers of DAPI-stained bodies present in diakinesis oocytes were assessed in intact adult hermaphrodites at day 1 of adulthood (24 hours post-L4, unless otherwise specified). Adults were fixed in Carnoy’s fixative solution (60% ethanol, 30% chloroform, and 10% glacial acetic acid) and stained with 4′,6-diamidino-2-phenylindole (DAPI) (10 mg/ml stock diluted 1:50,000 in 1X Phosphate-buffered saline; PBS) for 15 minutes in a humid chamber. Worms were mounted in Prolong Gold with DAPI, cured overnight at room temperature, and stored at 4 °C prior to imaging either on a Nikon A1r confocal microscope or Leica Stellaris 5 confocal with an integrated White Light Laser (WLL). Diakinesis images were procured as 0.2μm per plain Z-stacks and visualized using Volocity 3-D Imaging Software for Nikon images and LAS X for Leica images.

### γ-irradiation

Worms of specified genotypes were exposed to 10Gy of γ-irradiation using a ^137^Cs source (Gammacell1000 Elite; Nordion International). Analysis of diakinesis oocytes by DAPI staining was performed as described above at 28 h post-irradiation.

### Live imaging of *him-5* transgene

Day 1 adults of *dsb-1; eaIs15* and day-2 adults of, *dsb-2; eaIs15* were placed on glass slides and washed with M9 buffer (22 mM KH_2_PO_4_, 42 mM Na_2_HPO_4_, 85.5 mM NaCl, 1 mM MgSO_4_) to remove residual bacteria. Then the worms were incubated for 20 min with a solution of DRAQ5 (1:2000 in M9), washed for 1 minute with M9, and the gonads dissected in M9 with 0.1 μl of 1mM levamisole. Images were taken with a Leica Stellaris 5 confocal with a WLL.

### Yeast two-hybrid assay

*GAL4* activating domain– (pGAD-C1) and binding domain (pGBD-C1)–expressing vectors were co-transformed into the PJ69–4a yeast strain and selected by growth on SC-LEU-TRP medium. Three to five transformants were grown to early log phase to 0.2 OD_600_, and 5 μl were spotted onto medium to select for the plasmids (SC-LEU-TRP; loading control) or onto medium to select for expression of the reporter *HIS3* gene (SC-LEU-TRP-HIS; which is indicative of a yeast-two-hybrid interaction). Plates were incubated for 72 hours at 30°C and imaged. The experiments were done in triplicate and empty vectors in the activating and binding domains were used as negative controls.

### Co-Immunoprecipitation and Western Blot

In order to perform co-immunoprecipitation experiments, synchronized *GFP::dsb-1; him-5::3xHA* and *him-5::3xHA* or *him-17::3xHA; him-5::GFP::3xFLAG* and *him-5::GFP::3xFLAG* strains were grown until young adult stage (24h post-L4) and nuclear protein fractionation was executed as previously shown (Silva et al.; 2014). The chromatin-bound fraction was generated upon Benzonase digestion (25U/100 ml of extract for 1h at 4°C). 2 mg of nuclear extract (nuclear-soluble and chromatin-bound fractions were pooled together) was incubated with Agarose GFP traps (Chromotek) or anti-HA Affinity Matrix (Roche) in Buffer D (20mM HEPES pH 7.9, 150mM KCl, 20% glycerol, 0.2mM EDTA, 0.2% Triton X-100 and 1x complete Roche inhibitor) overnight at 4°C. The following day, the agarose beads were recovered by centrifugation at 7500 rpm for 2át 4°C and extensively washed with Buffer D at room temperature. After the final wash, the beads were resuspended in 40 ml of 2x Laemmli Buffer and boiled for 10´, after which they were spun at maximum speed for 1´ and the whole eluate was loaded on a precast 4–20% gradient acrylamide gel. Proteins were transferred on a nitrocellulose membrane for 90 minutes at 4°C at 100V and blocked for 1h at room temperature in 1x TBST containing 5% BSA. Mouse monoclonal anti-HA (Cell Signaling), chicken polyclonal anti-GFP (AbCam), mouse monoclonal anti-FLAG HRP-conjugated (Sigma), and polyclonal anti-XND-1 (Wagner et al., 2010) antibodies were diluted in blocking solution at 1:1000, 1:5000, 1:2000 and 1:2500 respectively and left to incubate overnight at 4°C. Washes were performed in 1x TBST and anti-mouse, anti-chicken, and anti-guinea pig HRP-conjugated secondary antibodies (ThermoFisher) were diluted 1:10,000 in 1x TBST containing 5% milk for 1h at room temperature. After several washes in 1xTBST, the membrane was incubated with Clarity Max ECL (BioRad) and imaged with a G-Box (Syngene).

### Immunofluorescence and STED imaging

Gonads were dissected from 1-day adults of *him-17::3xHA* worms in 1X sperm salts (50 mM PIPES pH 7.0, 25 mM KCl, 1 mM MgSO4, 45 mM NaCl, and 2 mM CaCl2) with 1 mM levamisole and fixed in 1% paraformaldehyde diluted in 1X sperm salts for 5 min in a humid chamber. Slides were then frozen on a metal block on dry ice for at least 10 min prior to flicking off the coverslip and immersing in 100% ethanol at −20° C for 2 min. Slides were then washed in PBSTB [1XPBS with 0.1% Tween and 0.1% bovine serum albumin (BSA)], and incubated overnight at 4°C with primary antibody: mouse anti-HA (Cell Signaling), guinea pig anti-XND-1(Wagner et al., 2010), and rabbit anti-HTP-3 (Das et al., 2022) (all diluted 1:2000 in PBST). The next day, slides were washed 3X in PBSTB for 10 min and incubated with secondary antibodies: anti-mouse Alexa 488 (Molecular Probes), anti-guinea pig 633, and goat anti-rabbit Alexa 568 (Invitrogen, all diluted 1:2000 in PBSTB) for 2 h at room temperature in the dark. Slides were then washed 2 X 10 min in PBSTB, mounted in Prolong Gold without DAPI (Invitrogen) and put in the dark to cure overnight before imaging. The images were taken using a Stellaris8 TauSTED equipped with three depletion lines.

### HIM-17 and mass spectrometry analysis

Preparation and freezing of synchronized worms were carried out as in Cheeseman et al., (2004) except that worms were grown on 8P plates seeded with the *E. coli* strain NA22 at room temperature. Worm lysis was carried out by grinding worm pellets in liquid nitrogen and subsequent sonication (Diagenode) was performed with the following settings: 3× 5 cycles on high with 30 seconds on and 30 seconds off. Lysate was centrifuged for 45 minutes at 15000 rpm at 4°C. To avoid unspecific protein binding the worm lysate was pre-cleared by incubation to uncoupled agarose beads (Chromotek, bab-20) for 1 hour at 4°C. Pre-cleared worm lysate was incubated with GFP-trap (Chromotek, gta-20) for 3 hours at 4°C. Beads were washed 3 times with lysis buffer containing proteinase inhibitors (Roche) and 7 times with lysis buffer without proteinase inhibitors. Proteins were eluted by adding 40 μl 2xLSB and boiling for 5 min at 95°C. Protein precipitation was carried out using ProteoExtract Protein Precipitation Kit (EMD Millipore, 539180–1KIT) according to the manufacturer’s protocol and submitted for MS analysis to the Taplin Biological Mass Spectrometry Facility.

## Supplementary Material

Supplement 1
