# Supplementary material for: Genetic and physical interactions reveal overlapping and distinct contributions to meiotic double-strand break formation in C. elegans
Source: bioRxiv. 2025 Aug 7:2024.02.23.581796. Originally published 2024 Feb 28. Preprint. [Version 3] doi: 10.1101/2024.02.23.581796 (PMC10925144; doi:10.1101/2024.02.23.581796)
Supplement: Supplementary file 1 [file NIHPP2024.02.23.581796V3-supplement-1.pdf]

SUPPLEMENTARY DATA

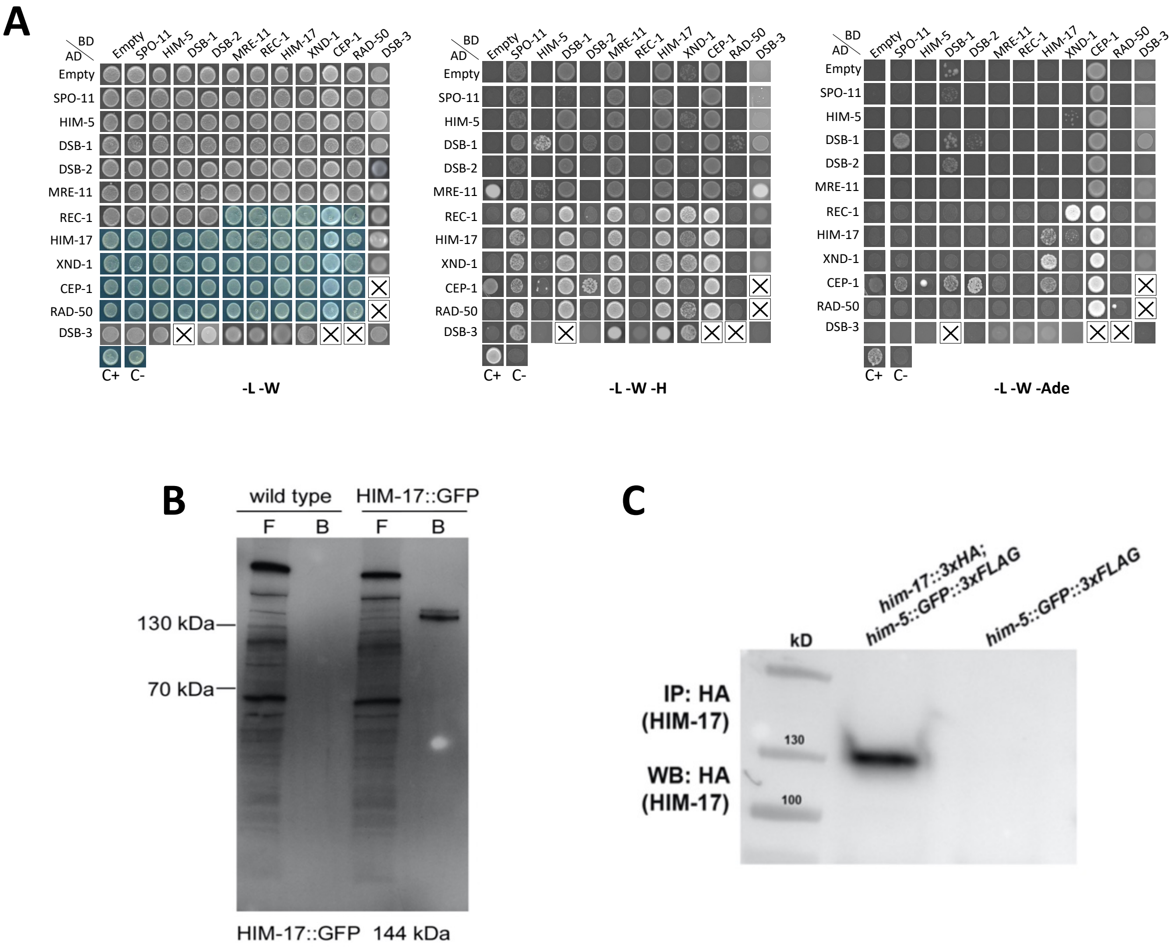

Figure S1: Yeast-2-Hybrid studies and control IP data. A) Representative Y2H interactions between DSB factors, as monitored by growth in different media: (left) -L-W used as a positive control; (center) -L-W-H showing weak interactions and, in some cases, self-activation; (right) -L-W -Ade showing strong interactions and self-activation by CEP-1. Every DSB factor was tested here as activating domain (AD) fusions as well as reciprocal DNA binding domain (BD) fusions. **B)** Western blot showing immunoprecipitation of HIM-17 from *him-17::GFP* whole worm lysates using anti-GFP antibody. Unbound protein fraction (flow through, F) and proteins bound to the GFP trap beads (**B**) from wild type and *him-17::GFP* transgenic worm lysates are shown. The HIM-17::GFP-specific signal is enriched by using a GFP trap. **C)** Western blot showing immunoprecipitation of HIM-17 from *him-17::3xHA;him-5::GFP::3xFLAG* whole worm lysates using an HA affinity matrix.

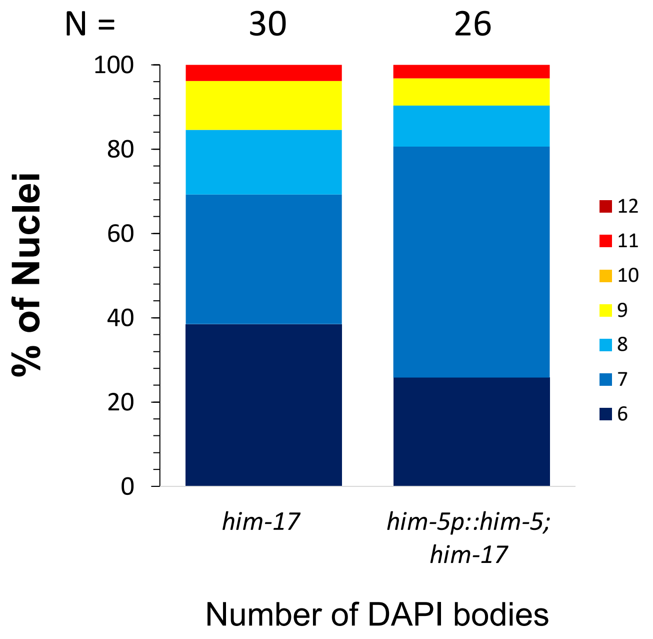

Figure S2: Quantification of DAPI-stained bodies at diakinesis in *him-17(ok424)* shows lack of rescue by HIM-5 expressed from its endogenous promoter. Colors correspond to the number of DAPI-stained bodies shown in the key. No statistical difference is observed.

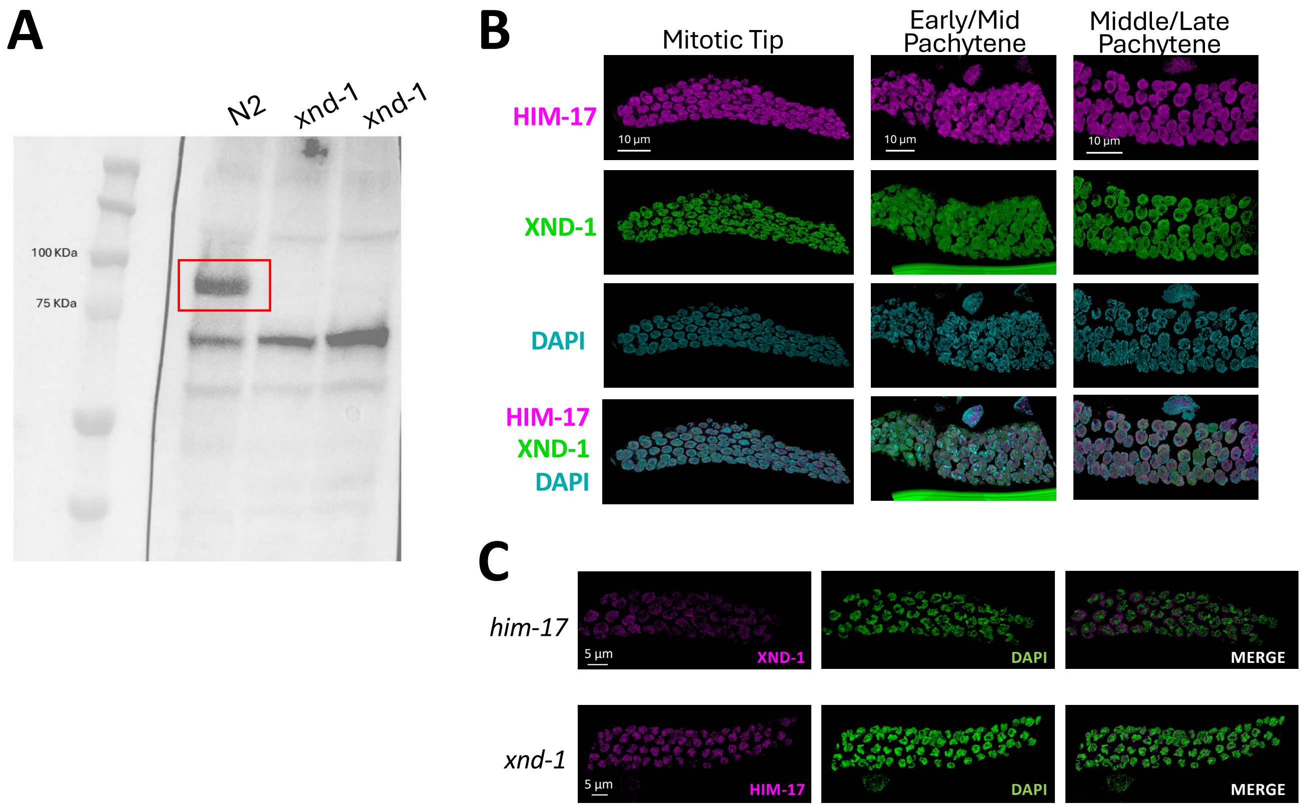

Figure S3: Localization of XND-1 and HIM-17 are non-overlapping and not interdependent. **A)** Western blot showing specificity of the guinea-pig anti-XND-1 antibody. Western analysis was performed on protein extracts from wild-type N2 worms and two independent *xnd-1* mutant strains. A distinct band corresponding to XND-1 (red square) is detected only in the N2 extract, confirming the specificity of the antibody. No signal is observed in the mutant strains, consistent with loss of *xnd-1* expression (see also Wagner et al., 2010). **B)** 3xHA::HIM-17 and anti-XND-1 staining do not over overlap with one another or with the DNA axes. Shown here are 3D renderings of confocal stacks from the mitotic zone, early-middle pachytene, and mid-late pachytene regions. **C)** Localization of XND-1 is normal in *him-17(ok424*$^{M-Z-}$*)* mutants (anti-XND-1, pink, DNA/DAPI, green) (top). Localization of 3xHA::HIM-17 is unaffected in *xnd-1(ok709)* mutants (anti-HA, pink; DNA/DAPI, green) (bottom).

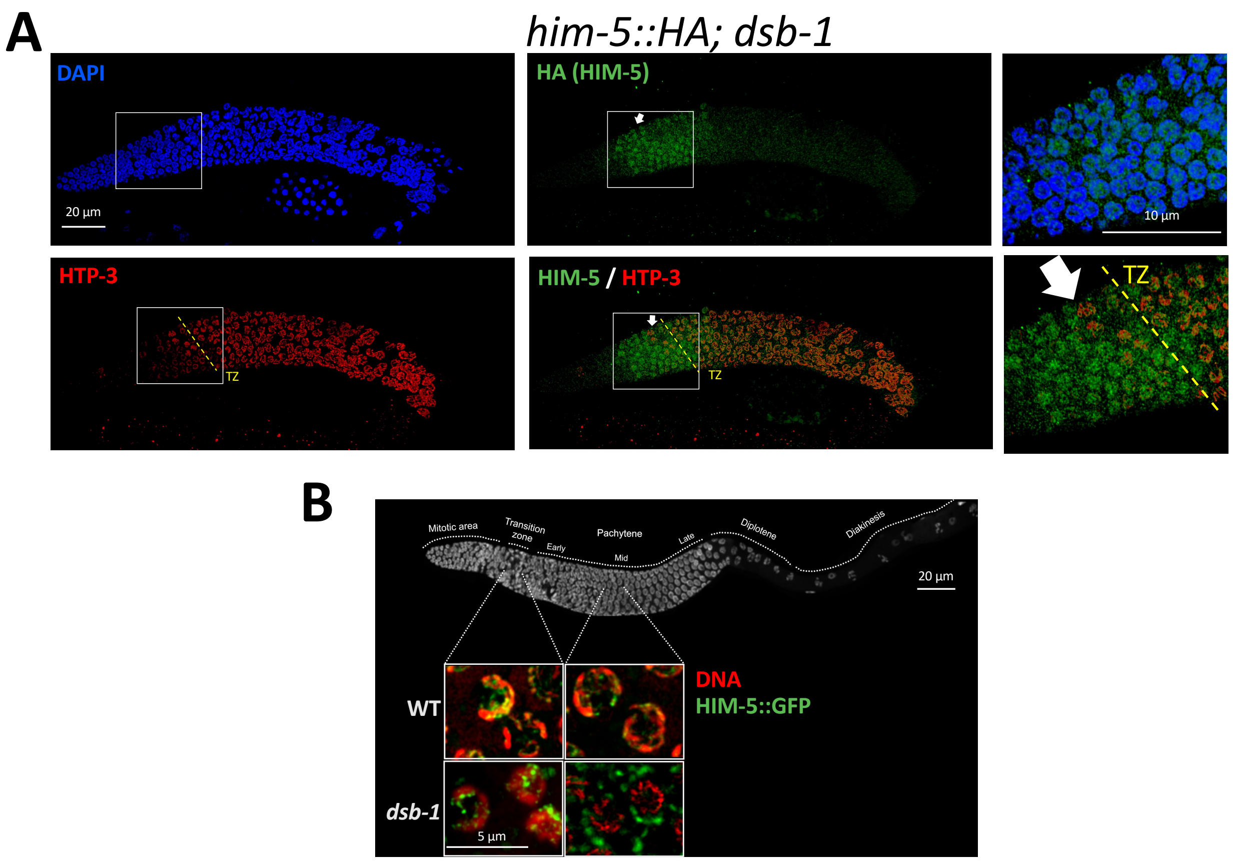

Figure S4. Localization of endogenous HIM-5 in *dsb-1* mutants.
A) Immunofluorescence analysis of HIM-5::HA in *dsb-1* mutants. DAPI (blue) stains DNA, anti-HA marks endogenously tagged HIM-5 (green), and HTP-3 (red) labels chromosome axes. HIM-5 appears localized in nuclei in pre-meiotic stages (indicated by arrows). However, after the transition zone (TZ), HIM-5 loses its nuclear localization. **B) Top:** *C. elegans* gonad fixed and stained with DAPI to show the organization and distribution of the nuclei along the Prophase I. **Bottom:** live imaging of nuclei in the transition zone (leptotene-zygotene) and middle-pachytene. *eaIs15 (Ppie-1::him-5::GFP*) is visualized in freshly dissected gonads by GFP fluorescence (green), and DNA by DRAQ5 (red). In *dsb-1* mutants, HIM-5 is nuclear in the transition zone and then only appears in cytoplasmic puncta by middle pachytene.

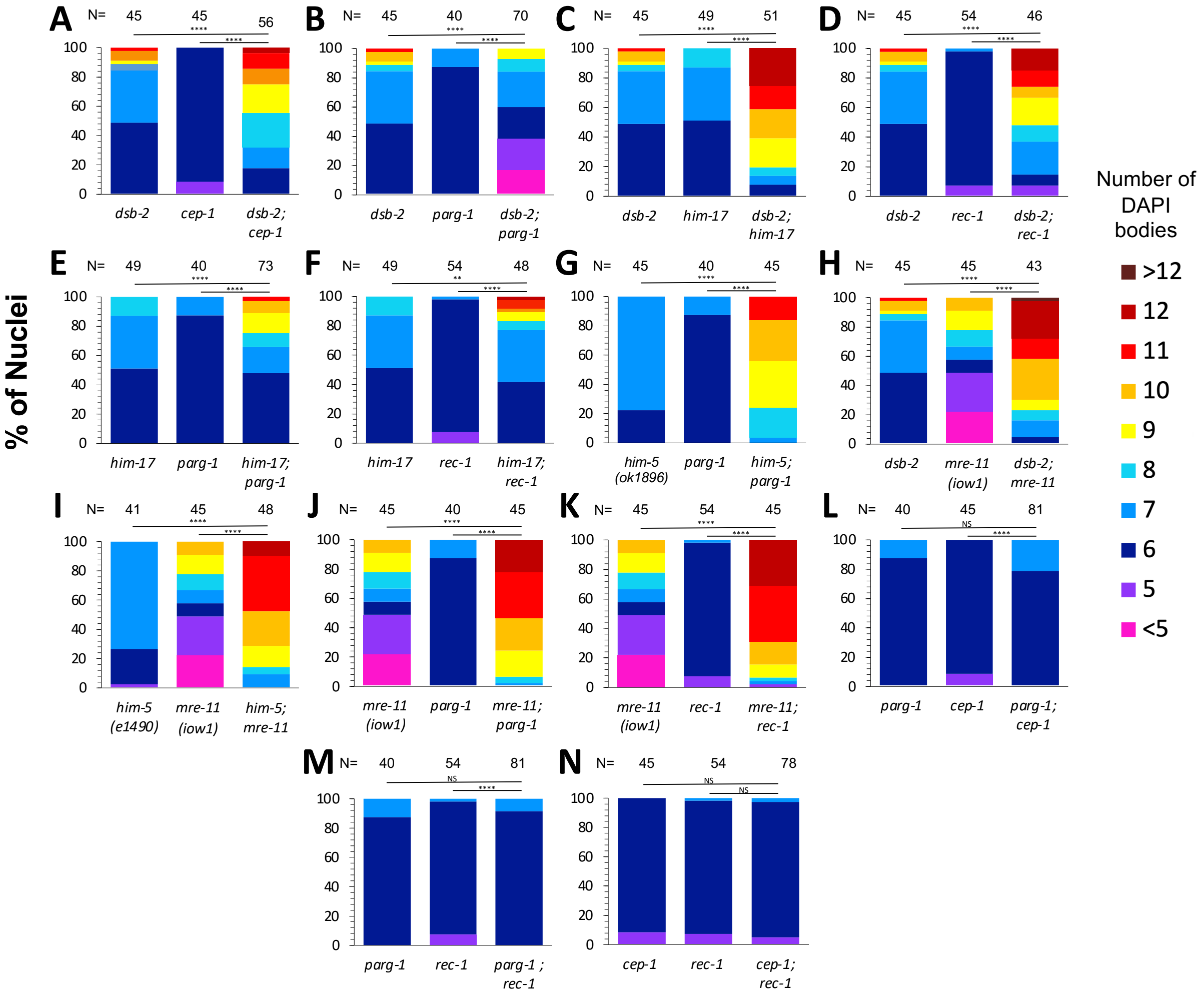

Figure S5. Epistasis analysis of DSB factors defines multiple genetic groups for crossover formation. A-N) Crossover formation is assessed by the number of DAPI-staining bodies at diakinesis. Each graph shows the quantification of DAPI-bodies in diakinesis nuclei for the indicated single and double mutants. Color indicates the number of DAPI-staining bodies. Sample sizes (N) are indicated. Statistical significance for comparisons between groups is shown at the top (NS= not significant, **p < 0.01, *** p<0.001, **** p< 0.0001).

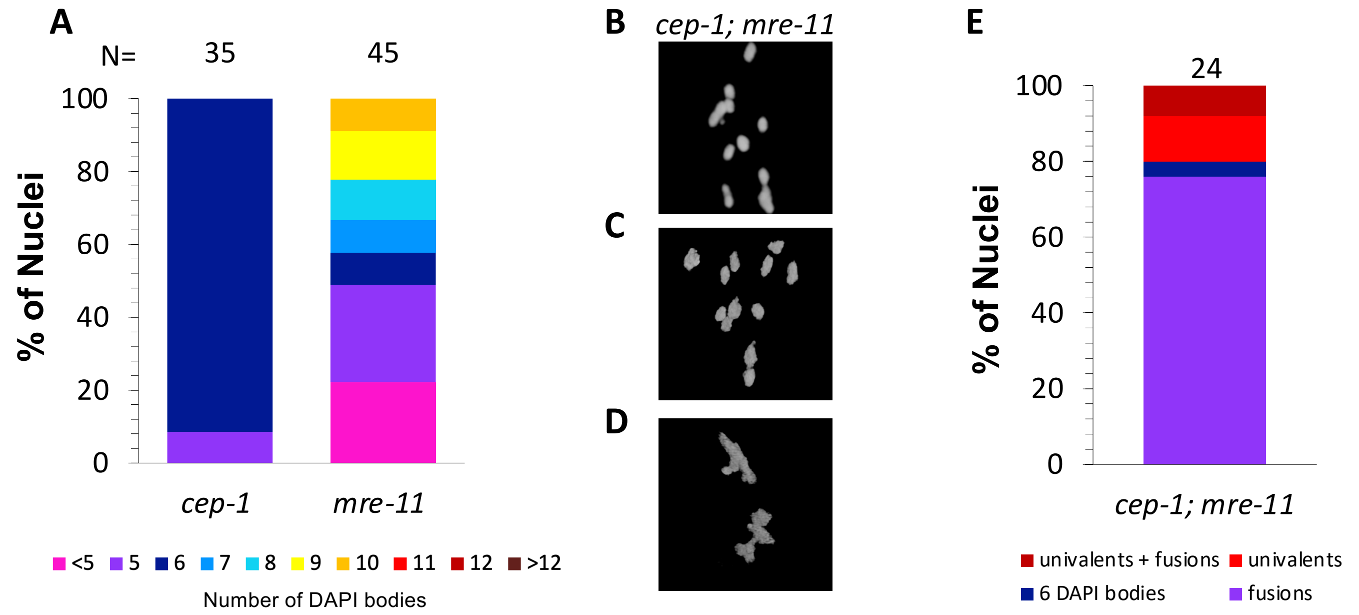

Figure S6: Mixed phenotypes are seen in *cep-1(lg12501); mre-11(iow1)* double mutants. A and E) Quantification of the number of DAPI-stained bodies at diakinesis for the indicated genotypes. Color indicates the number of DAPI-stained bodies. **B-D)** Representative DAPI-stained images of oocytes in diakinesis for *cep-1;mre-11* worms showing univalents (**B, C**) and fusions (**D**).

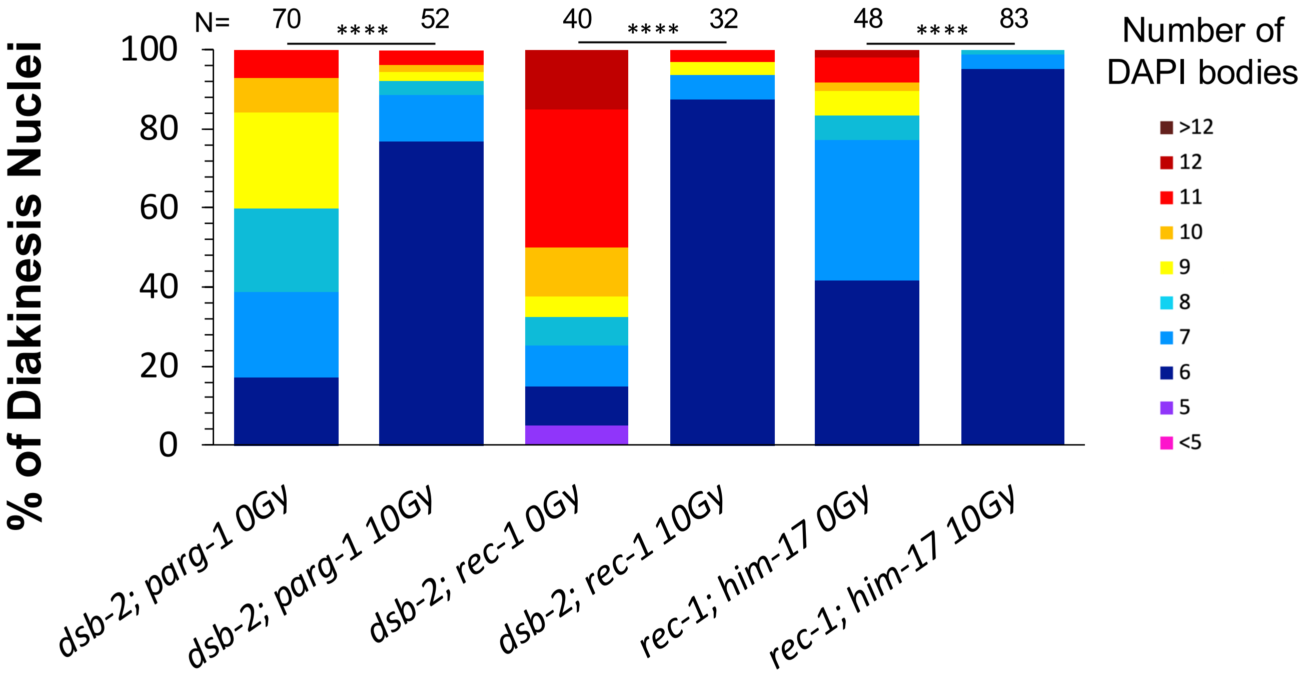

Figure S7: Irradiation rescues crossover defects of accessory factor double mutant strains. Quantification of DAPI-stained bodies at diakinesis for indicated genotypes with and without 10Gy of γ-irradiation. Color indicates the number of DAPI-staining bodies. Sample sizes (N) are indicated. Statistical significance for comparisons between groups is shown at the top (**** $p < 0.0001$).

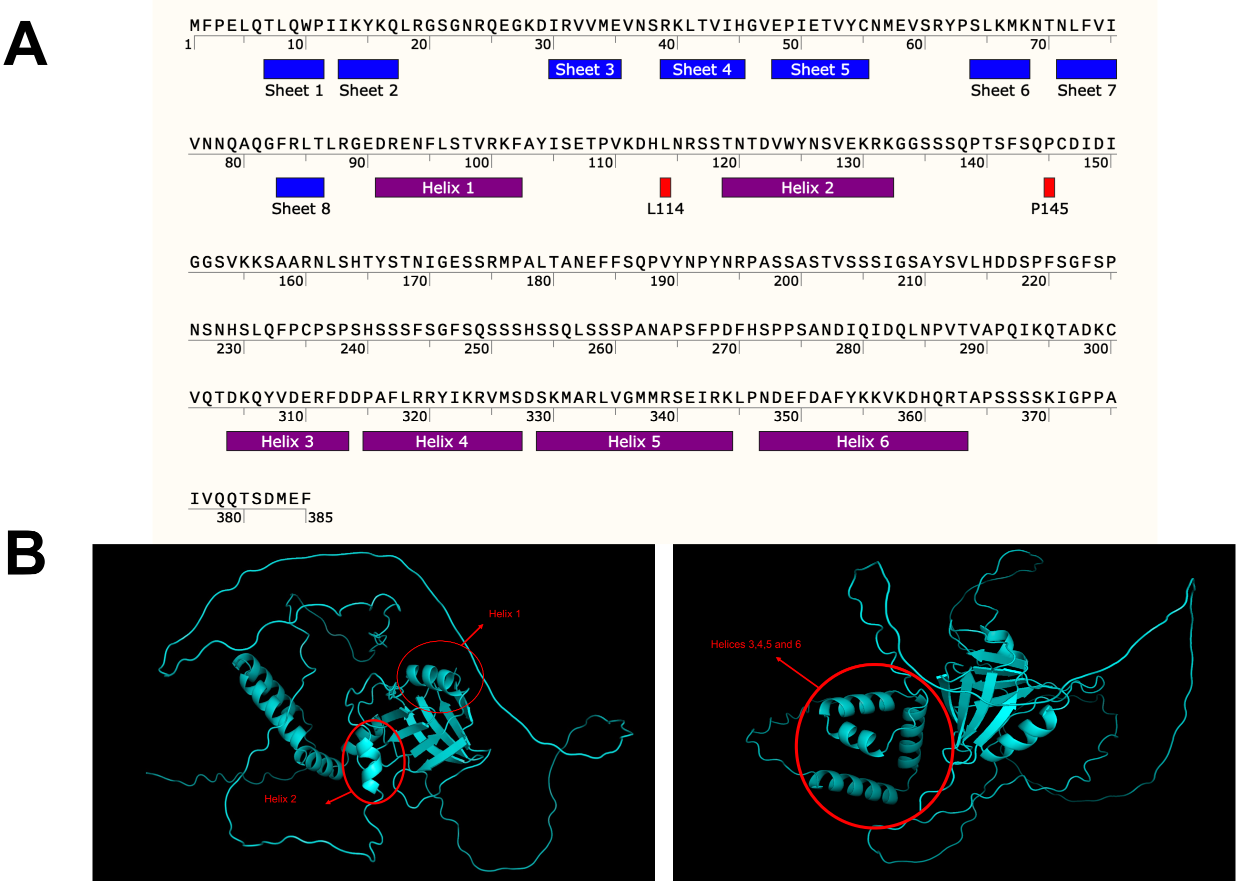

Figure S8: Structural analysis of DSB-1 identified potential interaction motifs. A) DSB-1 protein sequence with secondary structures demarcated: beta-sheets (blue); alpha-helices (purple). Deletions for Y2H assay shown in Figure 3 are marked with red and highlight in **B)** Alphafold2 model of DSB-1: helix 2 (left); helices 3-6 (right).

Strain	Genotype	Reference in text
N2	*C. elegans* var Bristol (N2).	Wild type
AV477	*dsb-2(me96)* II	*dsb-2*
AV280	*unc-119(e2498) III; him-17(ok424) V; meIs5[unc-119(+) + him-17::GFP].*	*him-17::GFP*
CA1117	*dsb-1(we11)* IV/*nT1[unc-?(n754) let-?]* (IV;V).	*dsb-1*
CB4088	*him-5(e1490)* V	*him-5(e1490)*
CB6036	*him-17(e2806)* V	*him-17*
KR5305	*rec-1(h2875)* I	*rec-1*
NSV250	*him-5(ddr43[him-5::3XHA])*	*him-5::3xHA*
NSV205	*him-17(ddr37[him-17::3XHA])* V	*him-17::3xHA*
NSV485	*eaIs4;him-17(ddr37[him-17::3XHA])* V	*him-17::3xHA;him-5::GFP::3xFLAG*
NSV508	*gfp::dsb-1;him-5::3xHA*	*gfp::dsb-1;him-5::3xHA*
QP1102	*rec-1(h2875)* I; *dsb-2(me96)* II	*dsb-2; rec-1*
QP1116	*mre-11(iow1), him-5(e1490)* V/ *nT1 [qIs51]* ((IV;V)	*him-5; mre-11*
QP1252	*rec-1(h2875);cep-1(lg12501)* I	*rec-1; cep-1*
QP1317	*rec-1 (h2875)* I;*him-17(e2806)/nT1g* V	*rec-1; him-17*
QP1366	*parg-1(gk120)* IV;*him-5(ok1896)* V	*him-5; parg-1*
QP 1367	*dsb-2(me96)* II;*parg-1(gk120)* IV	*dsb-2; parg-1*
QP1368	*rec-1(h2875)* I*;parg-1(gk120)* IV	*rec-1; parg-1*
QP1370	*cep-1(lg12501)* I*;parg-1(gk120)* IV	*parg-1; cep-1*
QP1373	*dsb-2(me96)* II;*him-17(e2806)* V	*dsb-2; him-17*
QP1374	*parg-1(gk120)* IV; *him-17(e2806)* V	*him-17; parg-1*
QP1550	*cep-1(lg12501)* I;*dsb-2(me96)* II	*cep-1; dsb-2*
QP1572	*parg-1(gk120)* IV;*mre-11(iow1)/nT1gU* [*unc-?(n754) let-?]* (IV; V)	*mre-11; parg-1*
QP1573	*cep-1(lg12501)* I;*mre-11(iow1)/ nT1[qIs51]* (IV;V)	*cep-1; mre-11*
QP1623	*rec-1(h2875)* I;*mre-11(iow1)/nT1gU [unc-?(n754) let-?]* (IV; V)	*mre-11; rec-1*
QP1624	*dsb-2(me96)* II;*mre-11(iow1)/ unc-?(n754) let-?* (IV; V)	*dsb-2; mre-11*
QP1710	*eaIs4[Phim-5::him-5::gfp::3xFLAG];him-17(e2707)* V	*eaIs4;him-17(e2707)*
QP1744	*eaIs4[Phim-5::him-5::gfp::3xFLAG + unc-119(+)];him-17(ok424)* V	*eaIs4; him-17(ok424)*
QP1749	*eaIs15[Ppie-1::him-5::gfp + unc-119(+)];him-17(ok424)* V	*eaIs15;him-17(ok424)*
QP1907	*dsb-1(we11) IV/nT1 [qIs51]* (IV; V);*eaIs15[Ppie-1::him-5::gfp +unc-119(+)]*	*dsb-1/nT1; eaIs15*
QP1909	*dsb-2(me96)* II;*eaIs15[Ppie-1::him-5::gfp +unc-119(+)];*	*dsb-2; eaIs15*
QP1961	*eaIs4 (Phim-5::him-5::gfp::3xFLAG::him-5 3' UTR + unc-119(+))*	*eaIs4*
PCM575	*dsb-1(icm97[GFP::dsb-1])* IV	*GFP::dsb-1*
RB869	*xnd-1(ok709)* III	*xnd-1*
RB1562	*him-5(ok1896)* V	*him-5(ok1896)*
SSM2	*mre-11(iow1)/nT1[qIs51]* (IV;V)	*mre-11*
VC130	*parg-1(gk120)* IV	*parg-1*
VC255	*+/nT1* IV*; him-17(ok424)/nT1* V	*him-17(ok424)*
XY1054	*cep-1(lg12501)*	*cep-1*

Table S1: Strains and genetics. All strains were derived from the wild-type Bristol strain N2 and were cultivated at 20 °C under standard conditions. Abbreviated names and full genotypes of the strains used in this study are listed here.

Protein ID	Protein name/ORF	Predicted Molecular Weight (kD)	Sequence Coverage (%) Replicate 1	Replicate 2
CE13489	HIM-17	111.0 (954 aa)	38.7%	42.2%
CE51355	CKU-80	80.7 (713 aa)	1.4%	29.5%
CE28985	MSH-6	133.6 (1186 aa)	8%	12.3%
CE44308	CKU-70	77.9 (679 aa)	13.7%	15.2%
CE30653	RPC-2	129.1 (1154 aa)	7.1%	9.8%
CE42531	F33H1.4	156.5 (1378 aa)	3.6%	4.4%
CE26863	XND-1	78.3 (702 aa)	13.7%	7.4%
CE02015	CID-1	162.6 (1425 aa)	2.3%	5.5%
CE04148*	KLP-10	77.3 (690 aa)	3.6%	7.7%
CE28437	LSY-2	42.3 (365 aa)	10.10%	9.9%
CE41449	MEL-46	110.5 (973 aa)	2.4%	2.5%
CE27382	TAG-153	80.5 (733 aa)	8.7%	8.9%
CE01045	SNFC-5	42.8 (381 aa)	6.8%	6%
CE28922	F26F4.12	25.6 (231 aa)	10.8%	10.8%
CE16631	KLP-19	123.1 (1083 aa)	3.4%	2.1%
CE36164	LSL-1	36.9 (318 aa)	6.9%	7.2%

*Listed as pseudogene in WormBase version: WS288

Table S2: Immunoprecipitation and Mass Spectrometry (IP-MS) Results of HIM-17::GFP from whole worm extracts. Sequence coverage refers to the percentage of the protein sequence that was pulled down in the HIM-17::GFP IP samples. Numbers are from two independent biological repeats and represent specific enrichment in HIM-17::GFP compared to control IPs (see Methods).
