# Supplementary material for: Genetic and physical interactions reveal overlapping and distinct contributions to meiotic double-strand break formation in C. elegans
Source: bioRxiv. 2024 May 30:2024.02.23.581796. Originally published 2024 Feb 28. Preprint. [Version 2] doi: 10.1101/2024.02.23.581796 (PMC10925144; doi:10.1101/2024.02.23.581796)
Supplement: Supplementary file 1 [file media-1.pdf]

## SUPPLEMENTARY DATA

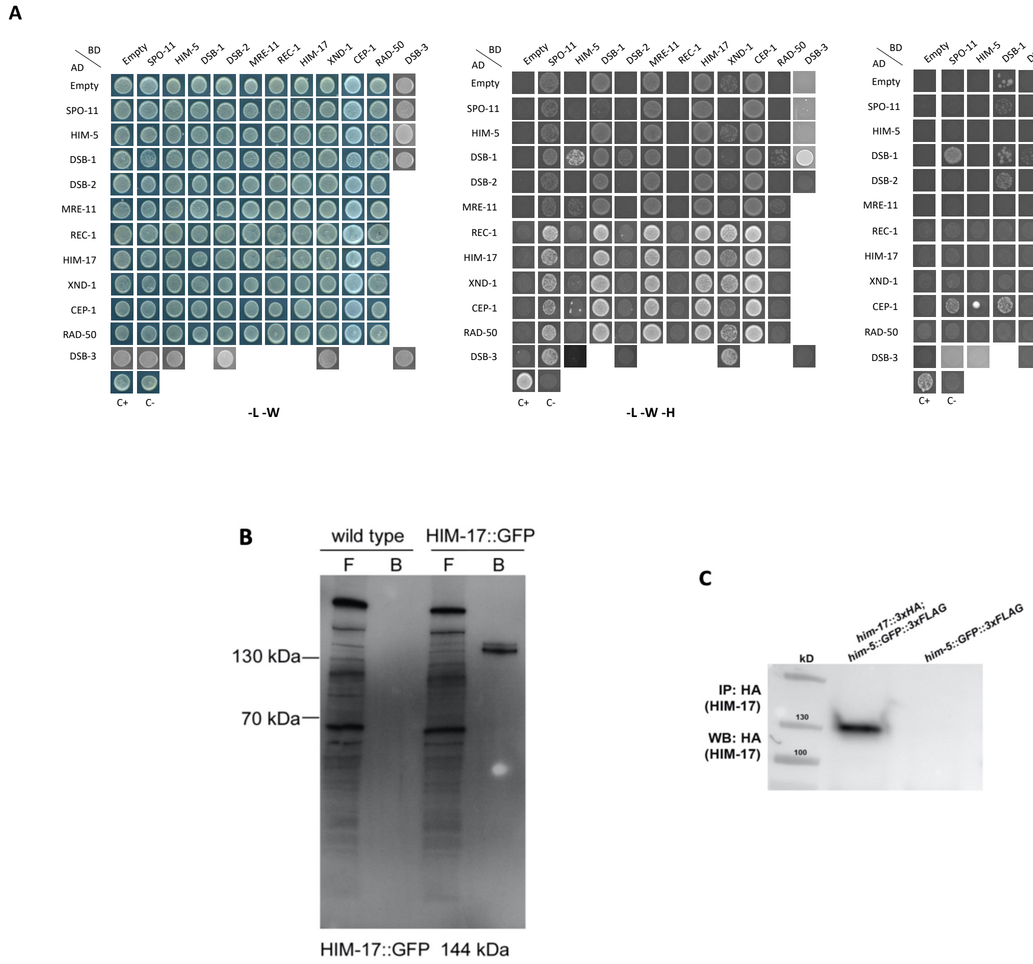


**Figure S1: Yeast-2-Hybrid studies and control IP data. A)** Representative Y2H interactions between DSB factors, as monitored by growth in different media: (left) -L-W used as a positive control; (center) -L-W-H showing weak interactions and, in some cases, self-activation; (right) -L-W- Ade showing strong interactions and self-activation by CEP-1. Every DSB factor was tested here as activating domain (AD) fusions as well as reciprocal DNA binding domain (BD) fusions. **B)** Western blot showing immunoprecipitation of HIM-17 from *him-17::GFP* whole worm lysates using an anti-HIM-17 antibody. Unbound protein fraction (flow through, F) and proteins bound to the GFP trap beads (B) from wild type and HIM-17::GFP transgenic worm lysates are shown. The HIM-17::GFP-specific signal is enriched by using a GFP trap. **C)** Western blot showing immunoprecipitation of HIM-17 from *him-17::3xHA;him-5::GFP::3xFLAG* whole worm lysates using an anti-HIM-17 antibody.

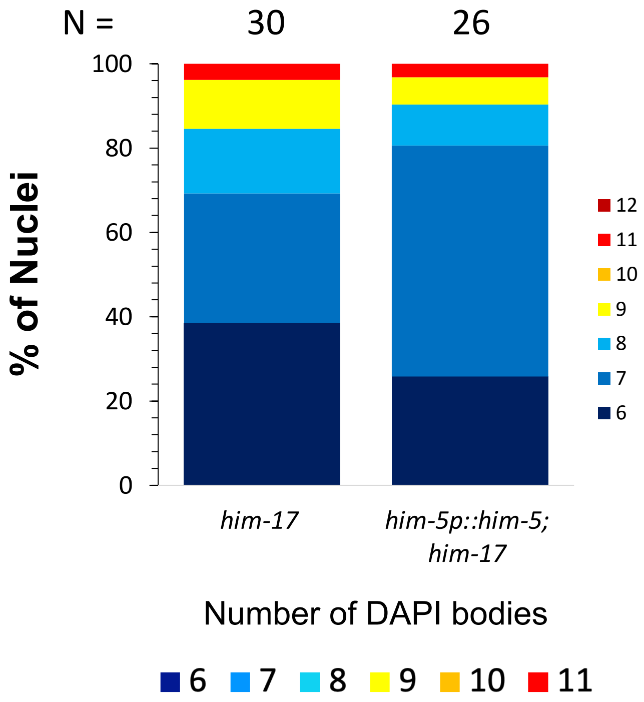


**Figure S2 : Quantification of DAPI-stained bodies at diakinesis shows lack of rescue by HIM-5 expressed from its endogenous promoter.** Colors correspond to the number of DAPI-stained bodies shown in the key below.

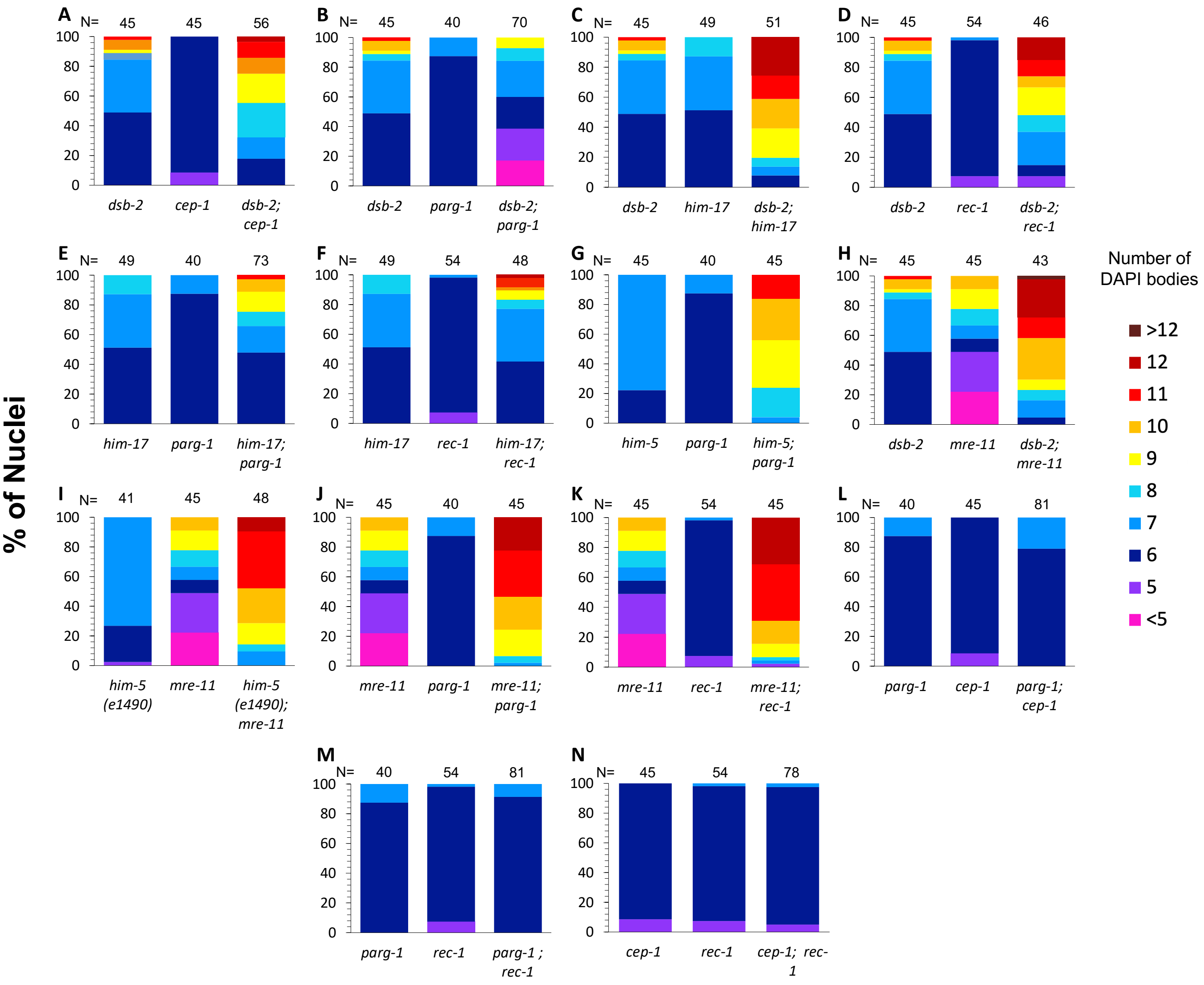


**Figure S3: Epistasis analysis of DSB factors defines multiple genetic groups for crossover formation. A-N)** Crossover formation is assessed by the number of DAPI-staining bodies at diakinesis**.** Each graph shows the quantification of DAPI-bodies in diakinesis nuclei for the indicated single and double mutants. Color indicates the number of DAPI-staining bodies. **O)** Genetic groups based on data presented here and previously published (Chung et al., 2015; Mateo et al., 2016; McClendon et al., 2016; Janisiw et al., 2020).

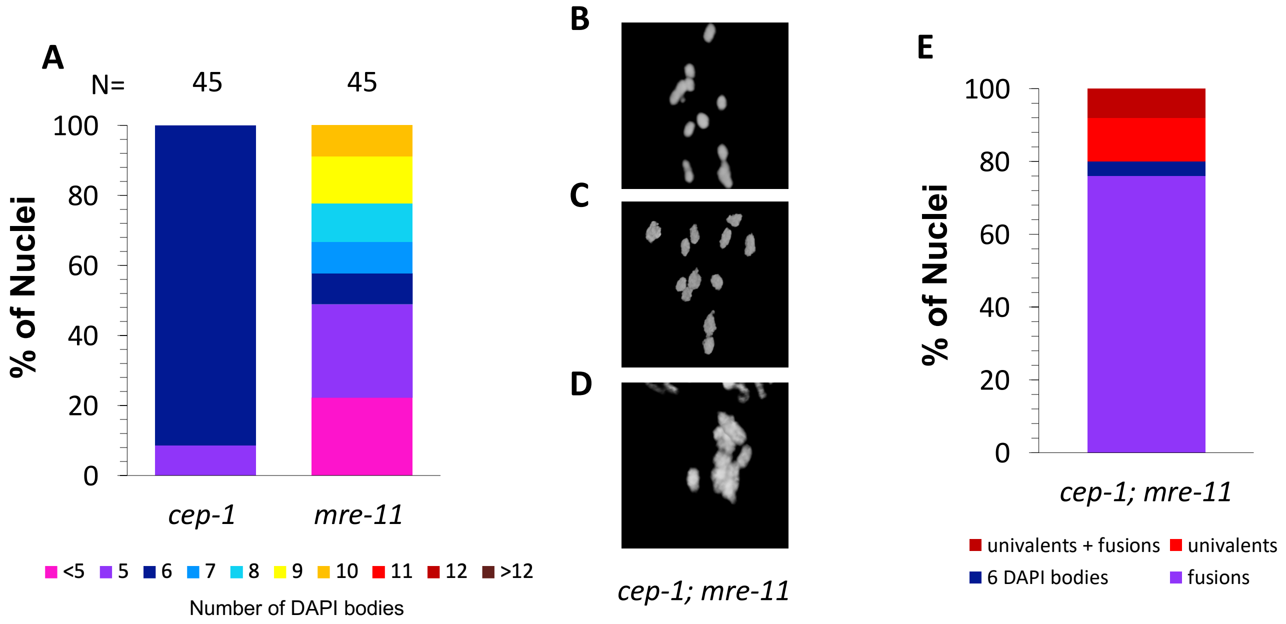


**Figure S4: Mixed phenotypes are seen in *cep-1(lg12501;mre-11(iow1)* double mutants. A and E)** Quantification of the number of DAPI-stained bodies at diakinesis for the indicated genotypes. Color indicates the number of DAPI-stained bodies**. B)** Representative DAPI-stained images of oocytes in diakinesis for *cep-1;mre-11* worms showing univalents (B, C) and fusions (D).

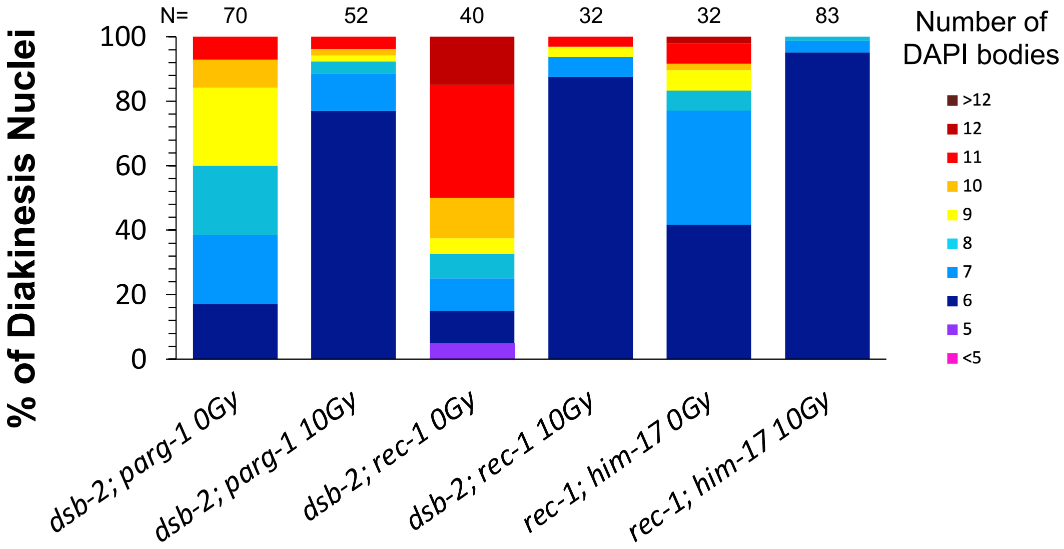


**Figure S5: Irradiation rescues to crossover defects of assessory factor double mutant strains.** Quantification of the number of DAPI-stained bodies at diakinesis for indicated genotypes with and without 10Gy of γ-irradiation. Color indicates the number of DAPI-staining bodies**.**

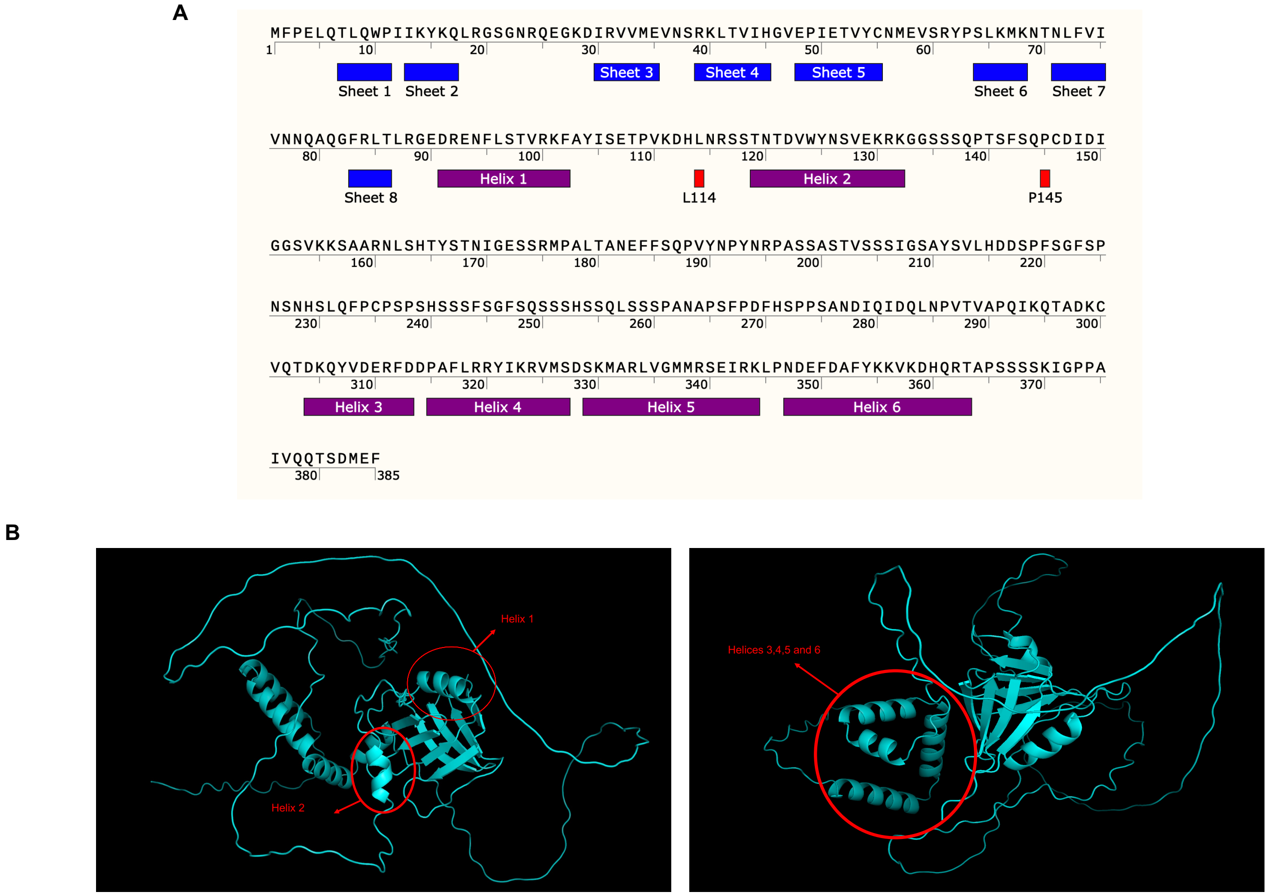


**Figure S6: Putative structural analysis of DSB-1 identified potential interaction motifs.** **A)** DSB-1 protein sequence. Secondary structures such as beta-sheets (blue) and alpha-helices (purple) are highlighted. In red are marked the sites where the deletions were introduced to create the truncated proteins used for yeast-two-hybrid in Figure 3. **B)** DSB-1 protein structure created by Alphafold2. Given that helixes are structures usually implicated in protein-protein interaction, some of them were deleted in the yeast-two-hybrid experiments shown in Figure 3. In this paper, we focused on the helix 2 and helices 3-6.

| Strain | Genotype | Reference in text |
|---|---|---|
| N2 | *C. elegans* var Bristol (N2). | Wild type |
| CA1117 | *dsb-1(we11)* IV/*nT1[unc-?(n754) let-?]* (IV;V). | *dsb-1* |
| AV477 | *dsb-2(me96)* II | *dsb-2* |
| RB1562 | *him-5(ok1896)* V | *him-5(ok)* |
| CB4088 | *him-5(e1490)* V | *him-5(e1490)* |
| CB6036 | *him-17(e2806)* V | *him-17* |
| KR5305 | *rec-1(h2875)* I | *rec-1* |
| VC130 | *parg-1(gk120)* IV | *parg-1* |
| SSM2 | *mre-11(iow1)/nT1[qIs51]* (IV;V) | *mre-11* |
| XY1054 | *cep-1(lg12501)* | *cep-1* |
| QP1368 | *rec-1(h2875)* I;*parg-1(gk120)* IV | *rec-1; parg-1* |
| QP1252 | *rec-1(h2875);cep-1(lg12501)* I | *rec-1; cep-1* |
| QP1370 | *cep-1(lg12501)* I;*parg-1(gk120)* IV | *parg-1; cep-1* |
| QP1317 | *rec-1 (h2875)* I;*him-17(e2806)/nT1g* V | *rec-1; him-17* |
| QP1102 | *rec-1(h2875)* I; *dsb-2(me96)* II | *dsb-2; rec-1* |
| QP 1367 | *dsb-2(me96)* II;*parg-1(gk120)* IV | *dsb-2; parg-1* |
| QP1373 | *dsb-2(me96)* II;*him-17(e2806)* V | *dsb-2; him-17* |
| QP1624 | *dsb-2(me96)* II;*mre-11(iow1)/ unc-?(n754) let-?* (IV; V) | *dsb-2; mre-11* |
| QP1550 | *cep-1(lg12501)* I;*dsb-2(me96)* II | *cep-1; dsb-2* |
| QP1573 | *cep-1(lg12501)* I;*mre-11(iow1)/ nT1[qIs51]* (IV;V) | *cep-1; mre-11* |
| QP1366 | *parg-1(gk120)* IV;*him-5(ok1896)* V | *him-5; parg-1* |
| QP1116 | *mre-11(iow1), him-5(e1490)* V/ *nT1 [qIs51]* ( (IV;V) | *him-5; mre-11* |
| QP1374 | *parg-1(gk120)* IV; *him-17(e2806)* V | *him-17; parg-1* |
| QP1572 | *parg-1(gk120)* IV;*mre-11(iow1)/nT1gU* [*unc-?(n754) let-?]* (IV; V) | *mre-11; parg-1* |
| QP1623 | *rec-1(h2875)* I;*mre-11(iow1)/nT1gU [unc-?(n754) let-?]* (IV; V) | *mre-11; rec-1* |
| QP1907 | *dsb-1(we11) IV/nT1 [qIs51]* (IV; V);*eaIs15[Ppie-1::him-5::gfp +unc-119(+)]* | *dsb-1/nT1; eaIs15* |
| QP1909 | *dsb-2(me96)* II;*eaIs15[Ppie-1::him-5::gfp +unc-119(+)];* | *dsb-2; eaIs15* |
| QP1749 | *him-17(ok424)* V;*eaIs15[Ppie-1::him-5::gfp + unc-119(+)];* | *eaIs15;him-17(ok424)* |

| | | |
|---|---|---|
| QP1744 | *him-17(ok424)* V;*eaIs4[Phim-5::him-5::gfp::3xFLAG + unc-119(+)]* | *eaIs4; him-17(ok424)* |
| PCM575 | *dsb-1(icm97[GFP::dsb-1])* IV | *GFP::dsb-1* |
| NSV250 | *him-5(ddr43[him-5::3XHA])* | *him-5::3xHA* |
| NSV205 | *him-17(ddr37[him-17::3XHA])* V | *him-17::3xHA* |
| NSV508 | *gfp::dsb-1;him-5::3xHA* | *GFP::dsb-1; him-5::3xHA* |
| QP?? | *Phim-5*::*him-5*::*GFP* | *eaIs4* |
| NSV485 | *eaIs4;him-17(ddr37[him-17::3XHA])* V | *him-17::3xHA; him-5::GFP::3xFLAG* |
| AV280 | *unc-119(e2498) III; him-17(ok424) V; meIs5[unc-119(+) + him-17::GFP].* | *him-17::GFP* |

**Table S1: Strains and genetics.** All strains were derived from the wild-type Bristol strain N2 and were cultivated at 20 °C under standard conditions. Abbreviated names and full genotypes of the strains used in this study are listed here.

| Protein ID | Protein name/ORF | Predicted Molecular Weight (kD) | Sequence Coverage (%) | |
|---|---|---|---|---|
| | | | Replicate 1 | Replicate 2 |
| CE13489 | HIM-17 | 111.0 (954 aa) | 38.7% | 42.2% |
| CE51355 | CKU-80 | 80.7 (713 aa) | 1.4% | 29.5% |
| CE28985 | MSH-6 | 133.6 (1186 aa) | 8% | 12.3% |
| CE44308 | CKU-70 | 77.9 (679 aa) | 13.7% | 15.2% |
| CE30653 | RPC-2 | 129.1 (1154 aa) | 7.1% | 9.8% |
| CE42531 | F33H1.4 | 156.5 (1378 aa) | 3.6% | 4.4% |
| CE26863 | XND-1 | 78.3 (702 aa) | 13.7% | 7.4% |
| CE02015 | CID-1 | 162.6 (1425 aa) | 2.3% | 5.5% |
| CE04148* | KLP-10 | 77.3 (690 aa) | 3.6% | 7.7% |
| CE28437 | LSY-2 | 42.3 (365 aa) | 10.10% | 9.9% |
| CE41449 | MEL-46 | 110.5 (973 aa) | 2.4% | 2.5% |
| CE27382 | TAG-153 | 80.5 (733 aa) | 8.7% | 8.9% |
| CE01045 | SNFC-5 | 42.8 (381 aa) | 6.8% | 6% |
| CE28922 | F26F4.12 | 25.6 (231 aa) | 10.8% | 10.8% |
| CE16631 | KLP-19 | 123.1 (1083 aa) | 3.4% | 2.1% |
| CE36164 | LSL-1 | 36.9 (318 aa) | 6.9% | 7.2% |

*Listed as pseudogene in WormBase version: WS288

**Table S2: Immunoprecipitation (IP) from HIM-17::GFP whole worm extracts with an antibody against GFP analyzed by mass spectrometry.** Sequence coverage refers to the percentage of the protein sequence that was pulled down in the HIM-17::GFP IP samples. Numbers are from two independent biological repeats.
