# Supplementary material for: Genetic and physical interactions reveal overlapping and distinct contributions to meiotic double-strand break formation in C. elegans
Source: bioRxiv. 2024 Feb 28:2024.02.23.581796. Preprint. [Version 1] doi: 10.1101/2024.02.23.581796 (PMC10925144; doi:10.1101/2024.02.23.581796)
Supplement: Supplementary file 1 [file NIHPP2024.02.23.581796v1-supplement-1.pdf]

SUPPLEMENTARY DATA

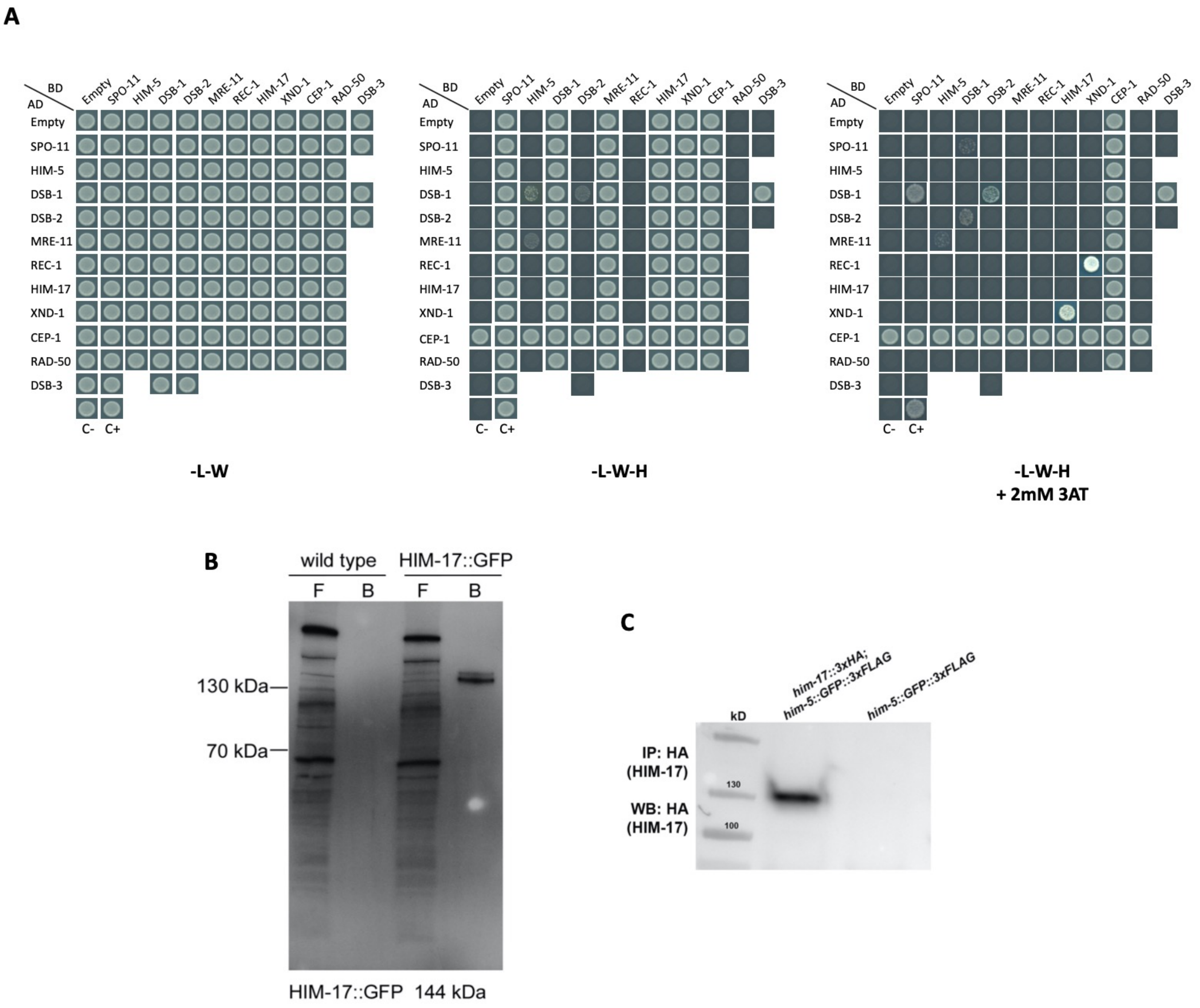

Figure S1: Yeast-2-Hybrid studies and control IP data. A) Representative Y2H interactions between DSB factors, as monitored by growth in different media: (left) -L-W used as a positive control; (center) -L-W-H showing weak interactions and, in some cases, self-activation; (right) -L-W-H + 2mM 3AT (competitive inhibitor) showing strong interactions and self-activation by CEP-1. Every DSB factor was tested here as activating domain (AD) fusions as well as reciprocal DNA binding domain (BD) fusions. **B)** Western blot showing immunoprecipitation of HIM-17 from *him-17::GFP* whole worm lysates using an anti-HIM-17 antibody. Unbound protein fraction (flow through, F) and proteins bound to the GFP trap beads (B) from wild type and HIM-17::GFP transgenic worm lysates are shown. The HIM-17::GFP-specific signal is enriched by using a GFP trap. **C)** Western blot showing immunoprecipitation of HIM-17 from *him-17::3xHA;him-5::GFP::3xFLAG* whole worm lysates using an anti-HIM-17 antibody.

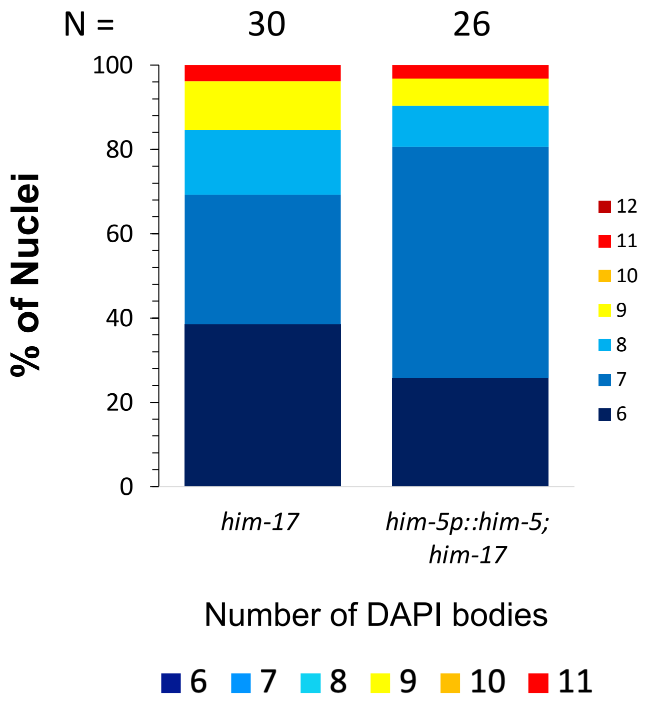

Figure S2 : Quantification of DAPI-stained bodies at diakinesis shows lack of rescue by HIM-5 expressed from its endogenous promoter. Colors correspond to the number of DAPI-stained bodies shown in the key below.

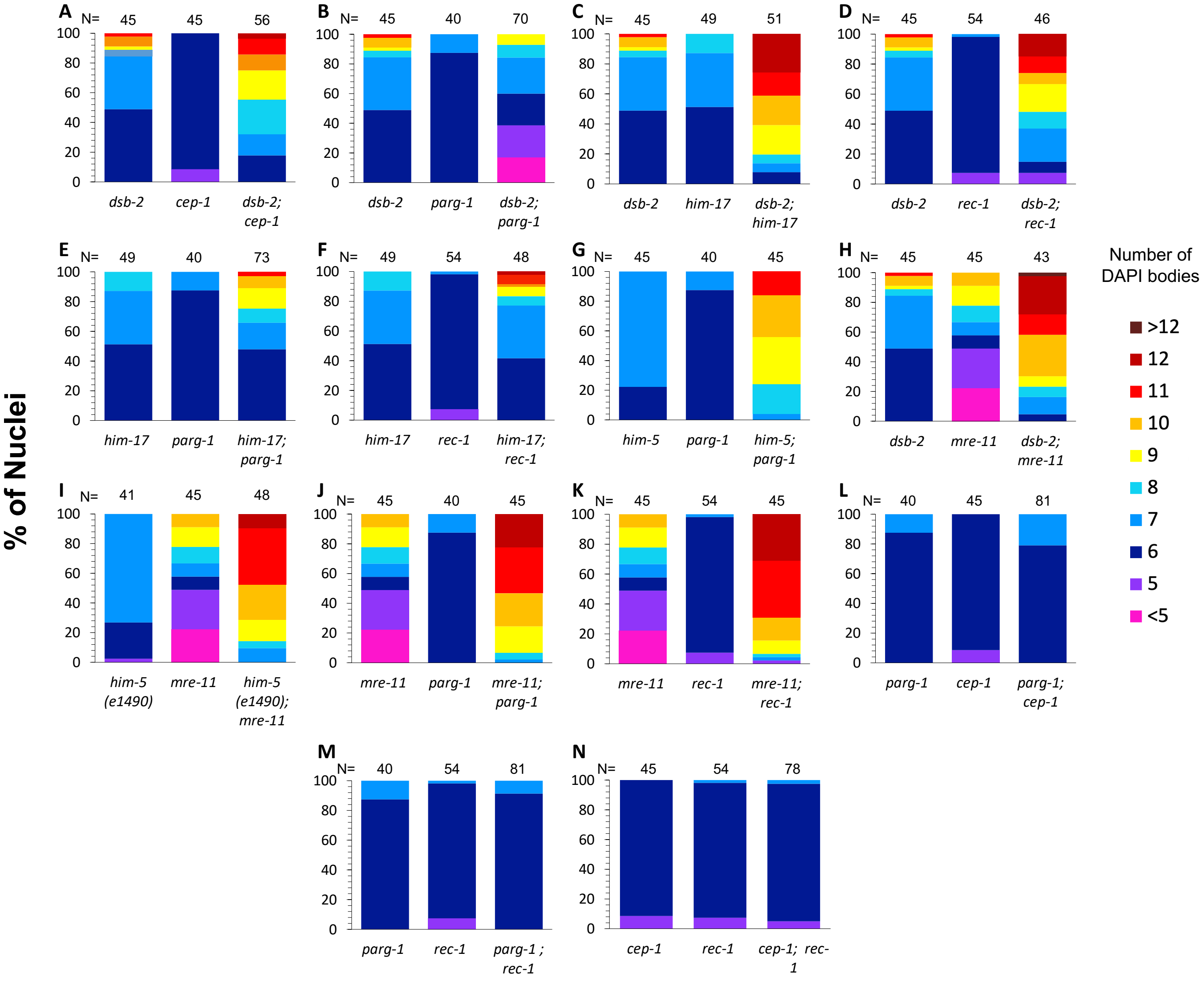

Figure S3: Epistasis analysis of DSB factors defines multiple genetic groups for crossover formation. **A-N)** Crossover formation is assessed by the number of DAPI-staining bodies at diakinesis**.** Each graph shows the quantification of DAPI-bodies in diakinesis nuclei for the indicated single and double mutants. Color indicates the number of DAPI-staining bodies. **O)** Genetic groups based on data presented here and previously published (Chung et al., 2015; Mateo et al., 2016; McClendon et al., 2016; Janisiw et al., 2020).

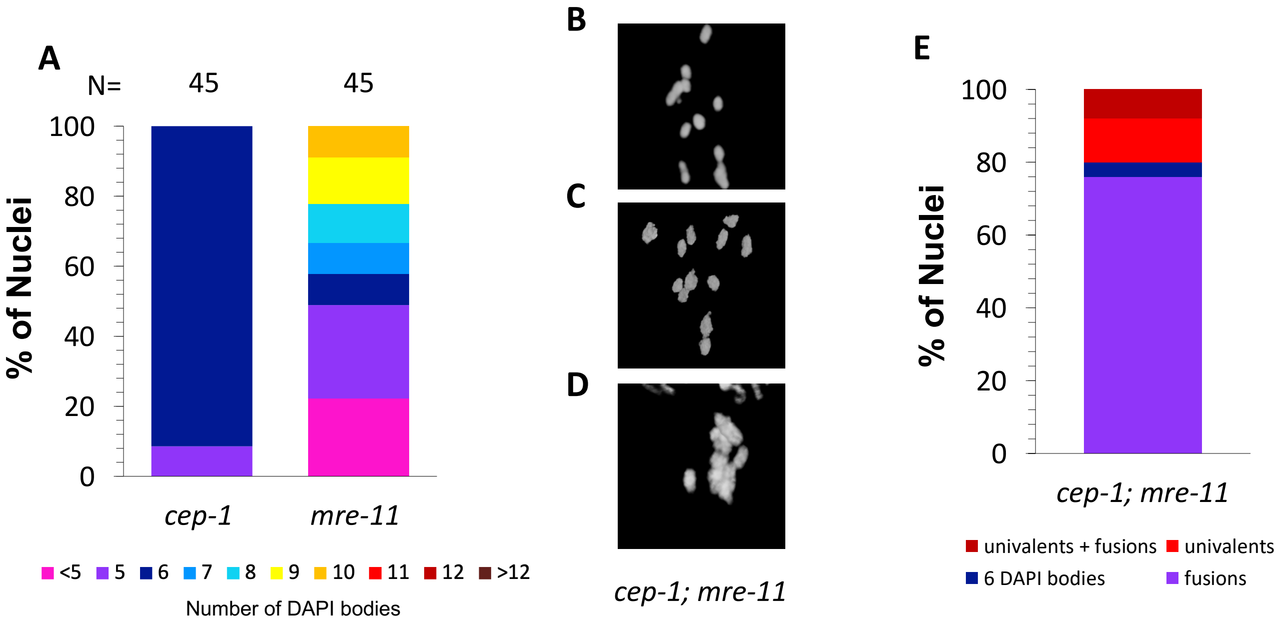

Figure S4: Mixed phenotypes are seen in *cep-1(lg12501;mre-11(iow1)* double mutants. A and E) Quantification of the number of DAPI-stained bodies at diakinesis for the indicated genotypes. Color indicates the number of DAPI-stained bodies**. B)** Representative DAPI-stained images of oocytes in diakinesis for *cep-1;mre-11* worms showing univalents (B, C) and fusions (D).

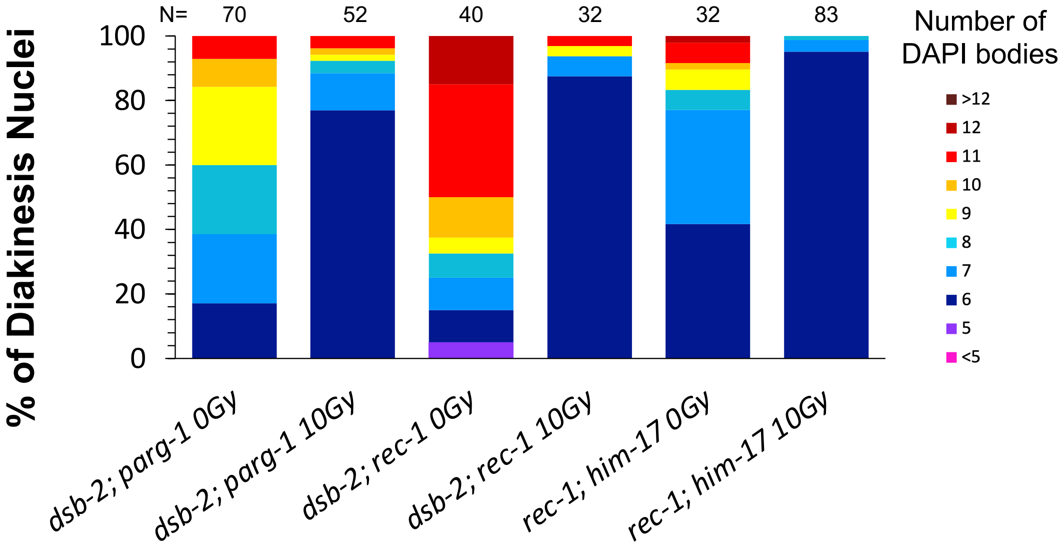

Figure S5: Irradiation rescues to crossover defects of assessory factor double mutant strains. Quantification of the number of DAPI-stained bodies at diakinesis for indicated genotypes with and without 10Gy of γ-irradiation. Color indicates the number of DAPI-staining bodies**.**

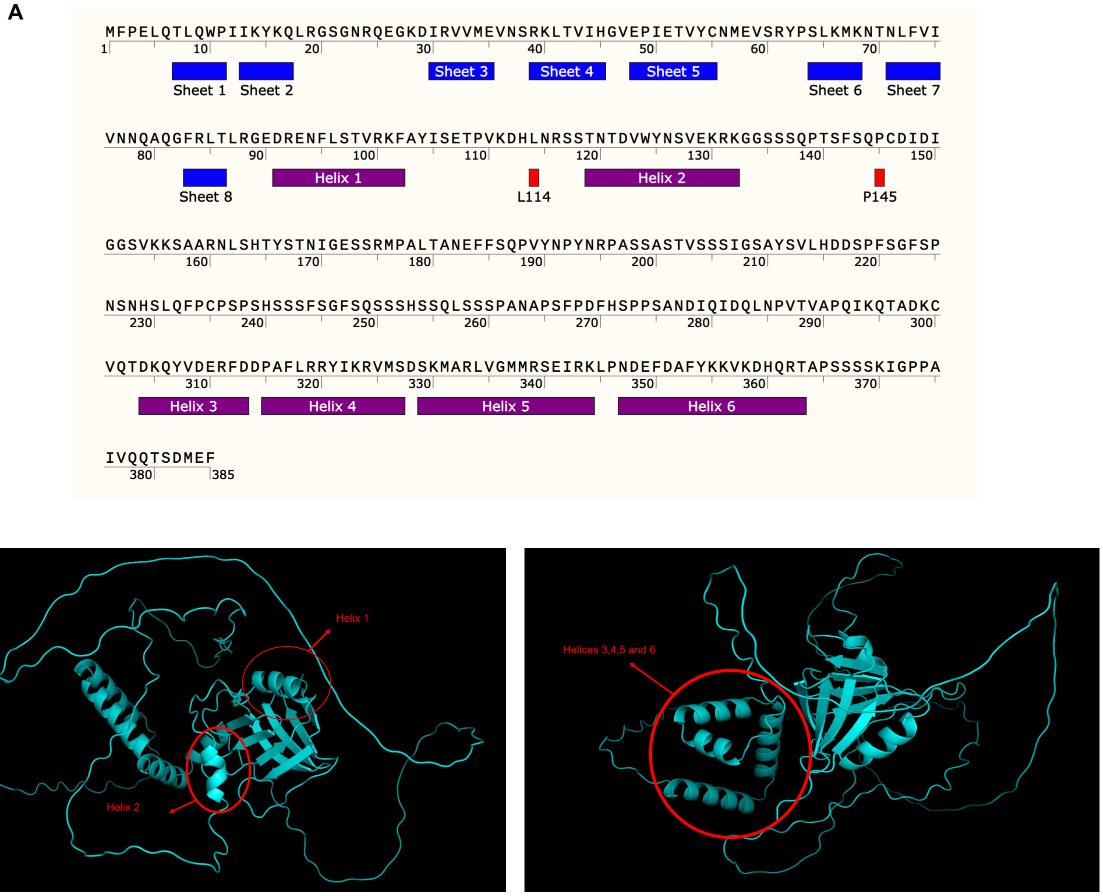

Figure S6: Putative structural analysis of DSB-1 identified potential interaction motifs. A) DSB-1 protein sequence. Secondary structures such as beta-sheets (blue) and alpha-helices (purple) are highlighted. In red are marked the sites where the deletions were introduced to create the truncated proteins used for yeast-two-hybrid in Figure 3. **B)** DSB-1 protein structure created by Alphafold2. Given that helixes are structures usually implicated in protein-protein interaction, some of them were deleted in the yeast-two-hybrid experiments shown in Figure 3. In this paper, we focused on the helix 2 and helices 3-6.

Strain	Genotype	Reference in text
N2	*C. elegans* var Bristol (N2).	Wild type
CA1117	*dsb-1(we11)* IV/*nT1[unc-?(n754) let-?]* (IV;V).	*dsb-1*
AV477	*dsb-2(me96)* II	*dsb-2*
RB1562	*him-5(ok1896)* V	*him-5(ok)*
CB4088	*him-5(e1490)* V	*him-5(e1490)*
CB6036	*him-17(e2806)* V	*him-17*
KR5305	*rec-1(h2875)* I	*rec-1*
VC130	*parg-1(gk120)* IV	*parg-1*
SSM2	*mre-11(iow1)/nT1[qIs51]* (IV;V)	*mre-11*
XY1054	*cep-1(lg12501)*	*cep-1*
QP1368	*rec-1(h2875)* I;*parg-1(gk120)* IV	*rec-1; parg-1*
QP1252	*rec-1(h2875);cep-1(lg12501)* I	*rec-1; cep-1*
QP1370	*cep-1(lg12501)* I;*parg-1(gk120)* IV	*parg-1; cep-1*
QP1317	*rec-1 (h2875)* I;*him-17(e2806)/nT1g* V	*rec-1; him-17*
QP1102	*rec-1(h2875)* I; *dsb-2(me96)* II	*dsb-2; rec-1*
QP 1367	*dsb-2(me96)* II;*parg-1(gk120)* IV	*dsb-2; parg-1*
QP1373	*dsb-2(me96)* II;*him-17(e2806)* V	*dsb-2; him-17*
QP1624	*dsb-2(me96)* II;*mre-11(iow1)/ unc-?(n754) let-?* (IV; V)	*dsb-2; mre-11*
QP1550	*cep-1(lg12501)* I;*dsb-2(me96)* II	*cep-1; dsb-2*
QP1573	*cep-1(lg12501)* I;*mre-11(iow1)/ nT1[qIs51]* (IV;V)	*cep-1; mre-11*
QP1366	*parg-1(gk120)* IV;*him-5(ok1896)* V	*him-5; parg-1*
QP1116	*mre-11(iow1), him-5(e1490)* V/ *nT1 [qIs51]* ((IV;V)	*him-5; mre-11*
QP1374	*parg-1(gk120)* IV; *him-17(e2806)* V	*him-17; parg-1*
QP1572	*parg-1(gk120)* IV;*mre-11(iow1)/nT1gU* [*unc-?(n754) let-?]* (IV; V)	*mre-11; parg-1*
QP1623	*rec-1(h2875)* I;*mre-11(iow1)/nT1gU [unc-?(n754) let-?]* (IV; V)	*mre-11; rec-1*
QP1907	*dsb-1(we11) IV/nT1 [qIs51]* (IV; V);*eaIs15[Ppie-1::him-5::gfp +unc-119(+)]*	*dsb-1/nT1; eaIs15*
QP1909	*dsb-2(me96)* II;*eaIs15[Ppie-1::him-5::gfp +unc-119(+)];*	*dsb-2; eaIs15*
QP1749	*him-17(ok424)* V;*eaIs15[Ppie-1::him-5::gfp + unc-119(+)];*	*eaIs15;him-17(ok424)*

QP1744	*him-17(ok424)* V;*eaIs4[Phim-5::him-5::gfp::3xFLAG + unc-119(+)]*	*eaIs4; him-17(ok424)*
PCM575	*dsb-1(icm97[GFP::dsb-1])* IV	*GFP::dsb-1*
NSV250	*him-5(ddr43[him-5::3XHA])*	*him-5::3xHA*
NSV205	*him-17(ddr37[him-17::3XHA])* V	*him-17::3xHA*
NSV508	*gfp::dsb-1;him-5::3xHA*	*GFP::dsb-1; him-5::3xHA*
QP??	*Phim-5::him-5::GFP*	*eaIs4*
NSV485	*eaIs4;him-17(ddr37[him-17::3XHA])* V	*him-17::3xHA; him-5::GFP::3xFLAG*
AV280	*unc-119(e2498) III; him-17(ok424) V; meIs5[unc-119(+) + him-17::GFP].*	*him-17::GFP*

Table S1: Strains and genetics. All strains were derived from the wild-type Bristol strain N2 and were cultivated at 20 °C under standard conditions. Abbreviated names and full genotypes of the strains used in this study are listed here.

Protein ID	Protein name/ORF	Predicted Molecular Weight (kD)	Sequence Coverage (%)	
			Replicate 1	Replicate 2
CE13489	HIM-17	111.0 (954 aa)	38.7%	42.2%
CE51355	CKU-80	80.7 (713 aa)	1.4%	29.5%
CE28985	MSH-6	133.6 (1186 aa)	8%	12.3%
CE44308	CKU-70	77.9 (679 aa)	13.7%	15.2%
CE30653	RPC-2	129.1 (1154 aa)	7.1%	9.8%
CE42531	F33H1.4	156.5 (1378 aa)	3.6%	4.4%
CE26863	XND-1	78.3 (702 aa)	13.7%	7.4%
CE02015	CID-1	162.6 (1425 aa)	2.3%	5.5%
CE04148*	KLP-10	77.3 (690 aa)	3.6%	7.7%
CE28437	LSY-2	42.3 (365 aa)	10.10%	9.9%
CE41449	MEL-46	110.5 (973 aa)	2.4%	2.5%
CE27382	TAG-153	80.5 (733 aa)	8.7%	8.9%
CE01045	SNFC-5	42.8 (381 aa)	6.8%	6%
CE28922	F26F4.12	25.6 (231 aa)	10.8%	10.8%
CE16631	KLP-19	123.1 (1083 aa)	3.4%	2.1%
CE36164	LSL-1	36.9 (318 aa)	6.9%	7.2%

*Listed as pseudogene in WormBase version: WS288

Table S2: Immunoprecipitation (IP) from HIM-17::GFP whole worm extracts with an antibody against GFP analyzed by mass spectrometry. Sequence coverage refers to the percentage of the protein sequence that was pulled down in the HIM-17::GFP IP samples. Numbers are from two independent biological repeats.
